# Pliocene-Quaternary crustal melting in central and northern Tibet and insights into crustal flow

**DOI:** 10.1038/ncomms11888

**Published:** 2016-06-16

**Authors:** Qiang Wang, Chris J. Hawkesworth, Derek Wyman, Sun-Lin Chung, Fu-Yuan Wu, Xian-Hua Li, Zheng-Xiang Li, Guo-Ning Gou, Xiu-Zheng Zhang, Gong-Jian Tang, Wei Dan, Lin Ma, Yan-Hui Dong

**Affiliations:** 1 State Key Laboratory of Isotope Geochemistry, Guangzhou Institute of Geochemistry, Chinese Academy of Sciences, Guangzhou 510640, China; 2 CAS Center for Excellence in Tibetan Plateau Earth Sciences (CETES), Beijing 100101, China; 3 School of Earth Sciences, University of Bristol, Wills Memorial Building, Queens Road, Bristol BS8 1RJ, UK; 4 Department of Earth and Environmental Sciences, University of St Andrews, North Street, St Andrews KY16 9AL, UK; 5 School of Geosciences, The University of Sydney, Sydney, New South Wales 2006, Australia; 6 Institute of Earth Sciences, Academia Sinica, Nangang, Taipei 11529, Taiwan; 7 Department of Geosciences, National Taiwan University, Taipei 10617, Taiwan; 8 Institute of Geology and Geophysics, Chinese Academy of Sciences, Beijing 100029, China; 9 ARC Centre of Excellence for Core to Crust Fluid Systems (CCFS) and the Institute for Geoscience Research (TIGeR), Department of Applied Geology, Curtin University, Perth, Western Australia 6845, Australia

## Abstract

There is considerable controversy over the nature of geophysically recognized low-velocity–high-conductivity zones (LV–HCZs) within the Tibetan crust, and their role in models for the development of the Tibetan Plateau. Here we report petrological and geochemical data on magmas erupted 4.7–0.3 Myr ago in central and northern Tibet, demonstrating that they were generated by partial melting of crustal rocks at temperatures of 700–1,050 °C and pressures of 0.5–1.5 GPa. Thus Pliocene-Quaternary melting of crustal rocks occurred at depths of 15–50 km in areas where the LV–HCZs have been recognized. This provides new petrological evidence that the LV–HCZs are sources of partial melt. It is inferred that crustal melting played a key role in triggering crustal weakening and outward crustal flow in the expansion of the Tibetan Plateau.

## Abstract (web summary)

The role of the low velocity-high conductivity zones (LV–HCZs) in developing the Tibetan Plateau has remained controversial. Here, Wang *et al.* present new geochemical and petrological data that show the LV–HCZs are sources of partial melt thus giving insight into the development of the Tibetan Plateau.

## Graphical abstract

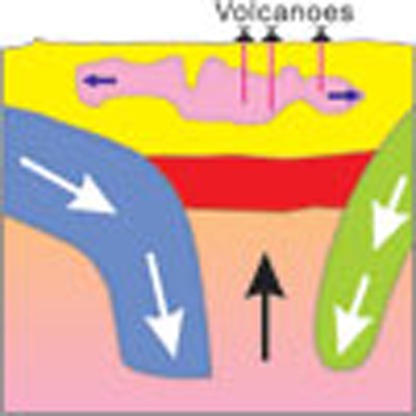

The Tibetan Plateau is an area of anomalously thick (∼50–90 km) continental crust and it is the highest and largest topographic feature on Earth^1^,^2^,^3^. Three main mechanisms have been proposed to account for crustal thickening and the development of the high topography: thinning of thickened mantle lithosphere^4^,^5^, intracontinental subduction^2^,^6^ and crustal (channel) flow^7^,^8^,^9^,^10^,^11^,^12^. The dispute stems partially from the sparse data on the thermal evolution of the Tibetan deep crust and mantle lithosphere, and inconclusive interpretations of the geophysically determined low-velocity zones (LVZs) and high-conductivity zones (HCZs) within the Tibetan crust. These LV–HCZs have been interpreted as weak layers within the crust that resulted from sub-horizontal orientation of mica crystals in a matrix of isotropic crystals^13^, the presence of mantle-derived melts^14^, aqueous fluid^15^,^16^, crustal shear zones^2^, and the presence of melt derived from within the zones^17^. In the crustal channel flow model, the LV–HCZs are considered to be partially molten layers within the crust based on geological evidence of anatectic melts^7^,^18^, numerical models^11^,^19^, and magnetotelluric (MT) and seismic models^1^,^8^,^20^,^21^,^22^. The diversity of models involving the LV–HCZs highlights that resolving their nature and origin requires petrological evidence on samples from the deep crust.

Volcanic rocks and entrained xenoliths derived from the deep crust or the upper mantle provide important information about the thermal regime of the Tibetan crust. It has been argued that the Tibetan crust is too dry to trigger crustal melting, based on granulite xenoliths entrained in Cenozoic volcanic rocks of central Tibet and a limited seismic wavespeed data set from central Tibet^23^. This caused some studies to call into question the role of crustal flow in the growth of the Tibetan Plateau^2^,^24^, and especially for the central and northern Tibetan Plateau^18^. However, other studies suggested that crustal melting played a key role in triggering crustal weakening and flow (refs 1, 8, 11, 20, 25, 26).

Here we report on 4.7–0.3 Myr ago old felsic volcanic rocks from the Qiangtang, the Songpan-Ganzi, and the Central Kunlun Blocks in central and northern Tibet, some of which contain granulite xenoliths. The volcanic rocks were predominantly generated by partial melting of mid-to-lower crustal rocks at temperatures of 700–1,050 °C and depths of 15–50 km, and as such they provide important new petrological evidence for the nature of LV–HCZs within the crust.

## Results

### Low-velocity–high-conductivity zones

**Figure 1:**
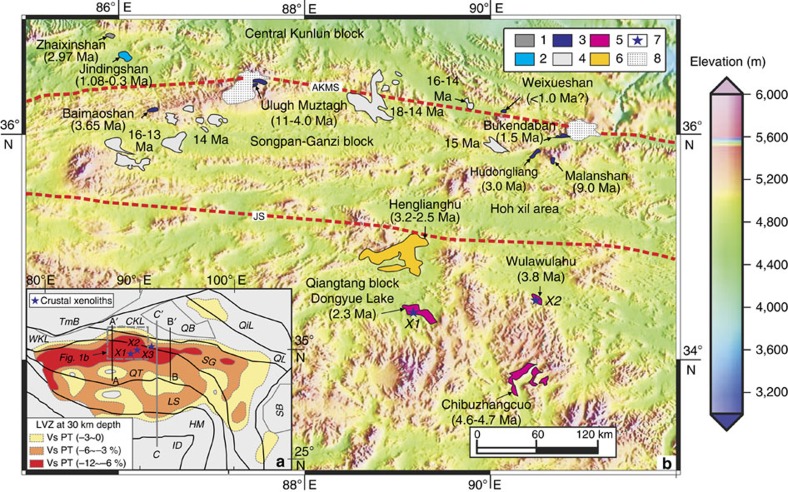
A topographic map of Tibet showing the tectonic blocks and the Late Cenozoic lavas and variations in crustal velocity. (**a**) The amplitude of the crustal low velocity zone (LVZ) Vs perturbation (PT) at 30-km depth beneath Tibet. This figure is modified from Yang *et al.*^22^ and Hacker *et al.*^17^. Lines A–A′ and B–B′ are similar to Seismic sections A–A′ and B–B′ of Figs 9 and 11 of Yang *et al.*^22^, and they delineate the locations of the vertical cross-sections shown in Fig. 4c. Line C–C′ is the line of the schematic section for Fig. 2. The tectonic units labelled are: CKL, Central Kunlun; HM, Himalaya; ID, India; LS, Lhasa; QB, Qaidam basin; QiL, Qilian; QL, Qinling; QT, Qiangtang; SB, Sichuan basin; SG, Songpan-Ganzi; TmB, Tarim basin; WKL, western Kunlun. X1, X2 and X3: crustal xenoliths locations. (**b**) A sketch map showing the locations of Late Cenozoic lavas and associated crustal xenoliths in central and northern Tibet. The original digital topography data for the central and northern Tibetan Plateau are from the Earth Resources Observation and Science (EROS) Center of United States Geological Survey (http://eros.usgs.gov/products/elevation/gtopo30.html). Main suture zones: AKMS, Anyimaqen-Kunlun-Muztagh; JS, Jinshajiang. Legends captions: (1) Pliocene (2.97 Myr ago) adakitic trachyandesites in the Zhaixinshan area (Supplementary Data set 1); (2) Quaternary (1.08–0.3 Myr ago) trachyandesites in the Jindingshan area (Supplementary Data set 1); (3) Miocene-Quaternary (11–1.5 Myr ago) rhyolites^34^,^35^,^36^ (Supplementary Data set 1); (4) Miocene (18–13 Myr ago) potassic–ultrapotassic lavas^5^,^31^; (5) Pliocene-Quaternary (4.7–2.3 Myr ago) non-adakitic felsic volcanic lavas in the central-northern Qiangtang Block (Supplementary Data set 1); (6) Pliocene (3.2–2.5 Myr ago) adakitic rhyolites in the Henglianghu area of the central-northern Qiangtang Block (Supplementary Data set 1); (7) crustal xenoliths; (8) glaciers. The data for crustal xenoliths (Locations X1, X2 and X3) from Cenozoic magmatic rocks are as follows: X1, granulite xenoliths from the Dongyue Lake area of the Qiangtang Block^23^; X2, Granulite xenoliths from the Wulanwulahu area of the Qiangtang Block (Supplementary Table 1); X3, Garnet-bearing mafic granulite and amphibolite xenoliths from 28 Myr ago intrusive rocks in the Hoh Xil area of Songpan-Ganzi (Supplementary Table 1).

**Figure 2:**
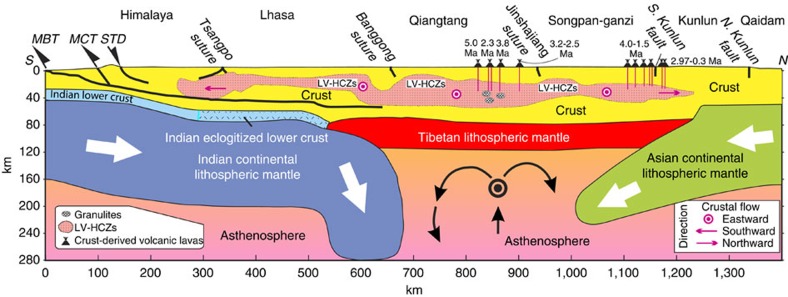
Integrated schematic cross-section across Tibet. This section illustrates one interpretation of the processes operating in the crust and mantle beneath Tibet (refs 1, 8, 15, 20, 21, 24, 28, 29). The Main Boundary Thrust Fault (MBT), the Main Central Thrust Fault (MCT), the Southern Tibet Detachment System (STD), the Tsangpo, Bangong, and Jinshajiang sutures, and the southern and northern Kunlun Faults are after Owens and Zandt^1^. The convection cell (black circles and arrows) underneath central and northern Tibet is superimposed eastwards (that is, from out of the plane in the figure) (after Tilmann *et al.*^28^). The low-velocity–high-conductivity zones (LV–HCZs) in the Tibetan crust are based on the geophysical data across the Tibetan Plateau (refs 1, 8, 15, 17, 20, 21, 22). The age data for Pliocene-Quaternary (4.7–0.3 Myr ago) felsic volcanic lavas are from Supplementary Data set 1.

A number of geophysical data sets indicate that LV–HCZs widely occur in the Tibetan crust (Fig. 1a) (refs 1, 8, 15, 16, 17, 20, 21, 22, 26, 27). Figure 2 presents an integrated schematic cross-section for the crust and mantle beneath the Tibetan Plateau based on the available geophysical data (refs 1, 8, 15, 20, 21, 24, 28, 29). This schematic section shows that the LV–HCZs occur at depths of 15–50 km, corresponding to the Tibetan mid-to-lower crust. Negative values for the amplitude of the LVZ (Vs perturbation (PT)=−12 to 0) are observed at depths of 15–50 km across most of Tibet, indicating that most of Tibet has Vs<3.4 km s^−1^ at those depths^17^,^22^ (Figs 1a and 2). However, the largest negative values (−12 to −6%) for the amplitude of the LVZ (Vs perturbation) (Fig. 1a) and the lowest shear wave speeds (Vs<3.35 or 3.25 km s^−1^) occur in central and northern Tibet^17^,^22^ (Figs 1a and 2). The MT data also record the lowest resistivities in the middle crust beneath the northern Qiangtang Block^14^,^15^,^21^,^26^,^27^ (Fig. 2).

### Cenozoic felsic volcanic lavas in central and northern Tibet

Cenozoic volcanic rocks occur widely on the Tibetan Plateau^30^. Eocene-Oligocene adakitic and alkalic to potassic–ultrapotassic mafic volcanic rocks outcrop in the Qiangtang Block in central Tibet^30^,^31^,^32^, Miocene adakitic and potassic–ultrapotassic mafic rocks are exposed in the Lhasa, the Songpan-Ganzi and the Central Kunlun Blocks of southern and northern Tibet and minor Pliocene-Quaternary potassic–ultrapotassic mafic rocks occur in the western Kunlun area^5^,^30^,^31^,^33^. Apart from minor 11–9 Myr ago rhyolites in the Ulugh Muztagh and Malanshan areas of northern Tibet^34^,^35^,^36^, felsic crustal melts appear to be confined to the 4.0–1.5 Myr ago rhyolites on the northern margin of Tibet^34^,^35^,^36^, and the 4.7–0.3 Myr ago rocks presented here (Supplementary Data set 1) (Figs 1b and 2). So far, no Miocene-Quaternary mantle-derived potassic–ultrapotassic mafic rocks have been recognized in the Qiangtang Block, but some Miocene (18–13 Myr ago) potassic–ultrapotassic mafic rocks occur in the Songpan-Ganzi and the Central Kunlun Blocks (Fig. 1b).

In the central-northern Qiangtang Block (Fig. 1b), the Dongyue Lake, Wulanwulahu, Chibuzhangcuo and Henglianghu volcanic rocks consist of dacites and minor trachyandesites and rhyolites. The samples studied have porphyritic textures with cryptocrystalline-glassy groundmass (Supplementary Fig. 1). Phenocrysts in the dacites and minor trachyandesites are of plagioclase, K-feldspar, quartz, titaniferous magnetite and minor sphene, and F-Ti-rich phlogopite (or biotite) and hastingsite. Phenocrysts in the rhyolites are mainly of plagioclase, K-feldspar and quartz, and their groundmass is dominated by glass and microcrystalline K-feldspar, ilmenite and titaniferous magnetite. In the north-central part of the Qiangtang Block (Fig. 1b), the volcanic rocks from the Dongyue Lake^23^ and the Wulanwulahu areas contain granulite xenoliths (Supplementary Table 1). These xenoliths mainly consist of orthopyroxene, spinel, plagioclase, K-feldspar, clinopyroxene, ilmenite and garnet±fluorinated biotite±fluorinated amphibole. In the Songpan-Ganzi and the Central Kunlun Blocks (Fig. 1b), at Bukendaban, Hudongliang, Weixueshan, Ulugh Muztagh and Baimaoshan, the young volcanic rocks are biotite- or two-mica-bearing rhyolites^34^,^35^,^36^ and the Jindingshan and Zhaixingshan volcanic rocks are trachyandesites (Fig. 1b; Supplementary Data set 1). The trachyandesites contain plagioclase, amphibole and K-feldspar phenocrysts and groundmass consisting of glass and microcrystalline plagioclase, amphibole and K-feldspar.

### Ages

**Figure 3:**
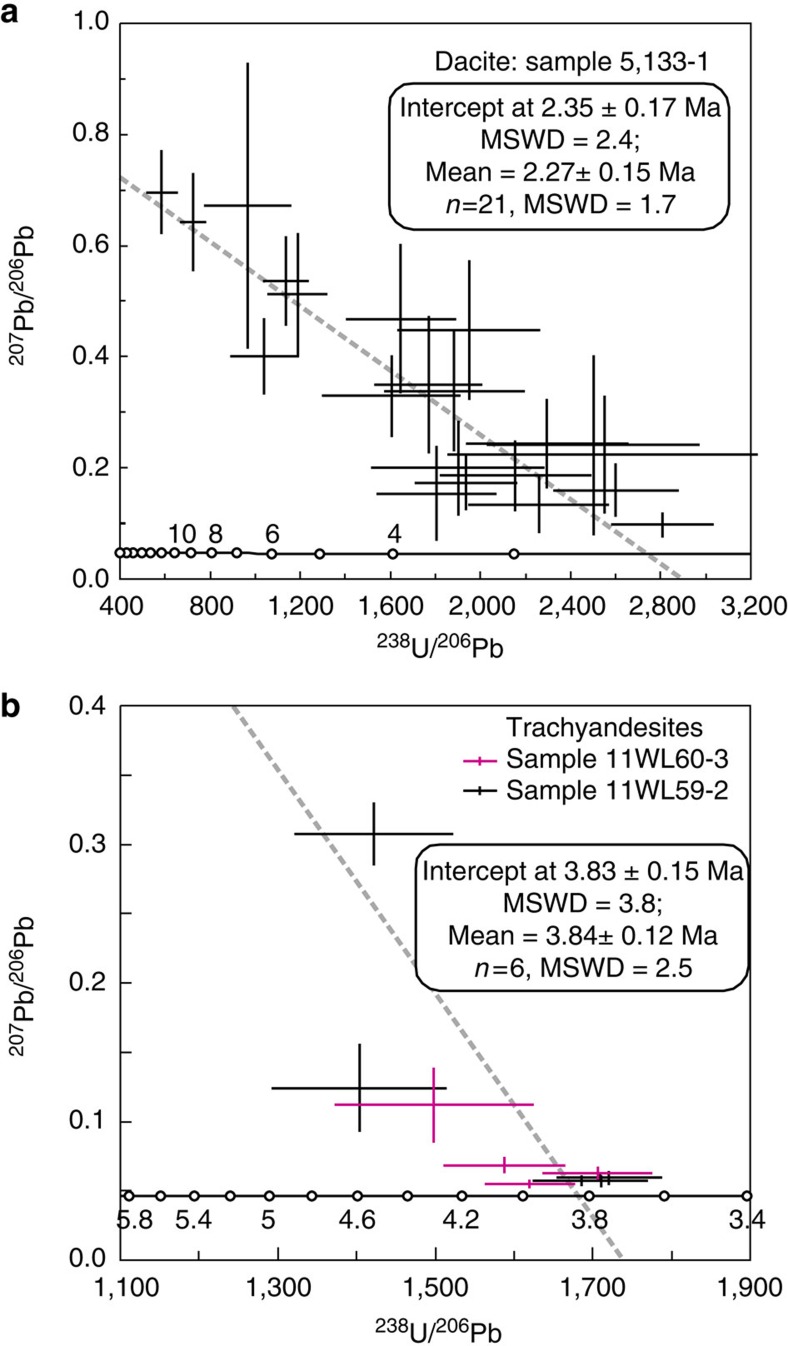
Zircon U-Pb Tera–Wasserburg plots for the zircon analyses. (**a**) The Dongyue Lake dacite from central-northern Qiangtang lavas. Sample 5133-1 has consistent zircon U-Pb lower intercept (2.35±0.17 Myr ago) and weighted mean (2.27±0.15 Myr ago) ages, indicating an eruption age of ∼2.3 Myr ago. (**b**) The Wulanwula trachyandesites from central-northern Qiangtang lavas. The red and black error bars are for samples 11WL60-3 and 11WL59-2, respectively. The circles on the horizontal line (part of the Tera–Wasserburg curve) in both **a** and **b** represent theoretically defined age scale values (in Myr ago). The analysed spots for two samples from approximately the same area (Supplementary Data set 1) exhibit consistent lower intercept (3.83±0.15 Myr ago) and weighted mean (3.84±0.12 Myr ago) ages, indicating an eruption age of ∼3.8 Myr ago. Error bars for the analytical age data are ‘s.e.'

**Figure 4:**
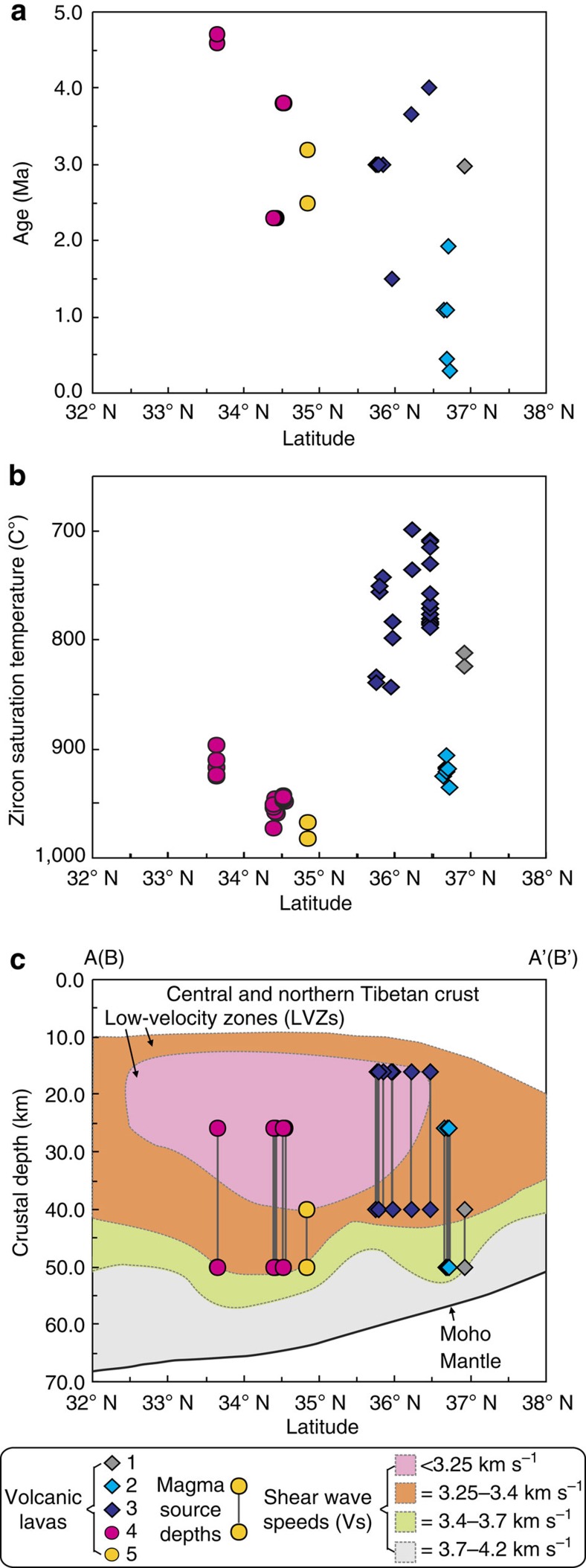
Variations in selected magma characteristics with latitude. (**a**) Plot of ages of volcanic lavas against latitude. The age data for the Pliocene-Quaternary felsic volcanic lavas of the central and northern Tibetan Plateau are from Supplementary Data set 1). (**b**) Plot of zircon saturation temperatures against latitude using the approach of Watson and Harrison^45^ (data summarized in Supplementary Data set 1. (**c**) The estimated depths of the magma sources plotted against latitude. The estimated crustal depths are summarized in Supplementary Data set 1. The sketched range for the low-velocity zones (LVZs) in the Tibetan crust is based on the geophysical data across the central and northern Tibetan Plateau (refs 1, 8, 14, 15, 17, 20, 21, 22), and the vertical cross-sections of shear wave speeds in absolute units (*V*_s_) along the A–A′ and B–B′ profiles (Fig. 1a) are from Yang *et al.*^22^ and Hacker *et al.*^17^. The symbols are as follows: 1, Pliocene (2.97 Myr ago) adakitic trachyandesites in the Zhaixinshan area of the Central Kunlun Block; 2, Quaternary (1.08–0.3 Myr ago) non-adakitic trachyandesites in the Jindingshan area of the Central Kunlun Block; 3, Pliocene-Quaternary (4.0–1.5 Myr ago) rhyolites in the Songpan-Ganzi Block; 4, Pliocene-Quaternary (4.7–2.3 Myr ago) non-adakitic felsic volcanic lavas in the central-northern Qiangtang Block; 5, Pliocene (3.2–2.5 Myr ago) adakitic rhyolites in the Henglianghu area of the central-northern Qiangtang Block.

Zircon U-Pb, and whole-rock and biotite ^40^Ar-^39^Ar and K-Ar ages indicate that the Pliocene-Quaternary lavas reported here were generated at 4.7–0.3 Myr ago (Fig. 1b; Supplementary Data set 1 and Supplementary Table 2). The lavas in the central-northern Qiangtang area yield ages of 4.7–2.3 Myr ago. The Dongyue Lake dacites have whole-rock and feldspar ^40^Ar/^39^Ar ages of∼3.0 Myr ago^23^, and CASIMS (Cameca IMS-1280) zircon U-Pb lower intercept and weighted mean ages of 2.35±0.17 Myr ago and 2.27±0.15 Myr ago, respectively (Fig. 3a) (Supplementary Table 2), indicating an eruption age of ∼2.3 Myr ago. They are therefore the youngest Cenozoic magmatic rocks dated from the central-northern Qiangtang Block. The Wulanwula trachyandesites have CASIMS zircon U-Pb lower intercept and weighted mean ages of 3.83±0.15 Myr ago and 3.84±0.12 Myr ago, respectively (Fig. 3b) (Supplementary Table 2), indicating an eruption age of ∼3.8 Myr ago. The lavas in the Songpan-Ganzi and the Central Kunlun areas were generated at 4.0–0.3 Myr ago (Fig. 1b and Supplementary Data set 1), and the Jingdingshan lavas are the youngest and have K-Ar ages of 0.45–0.30 Myr ago (Supplementary Data set 1). Overall the Pliocene-Quaternary lavas become younger northwards from central-northern Qiangtang to the Songpan-Ganzi and the Central Kunlun areas (Figs 2 and 4a).

### Geochemistry

**Figure 5:**
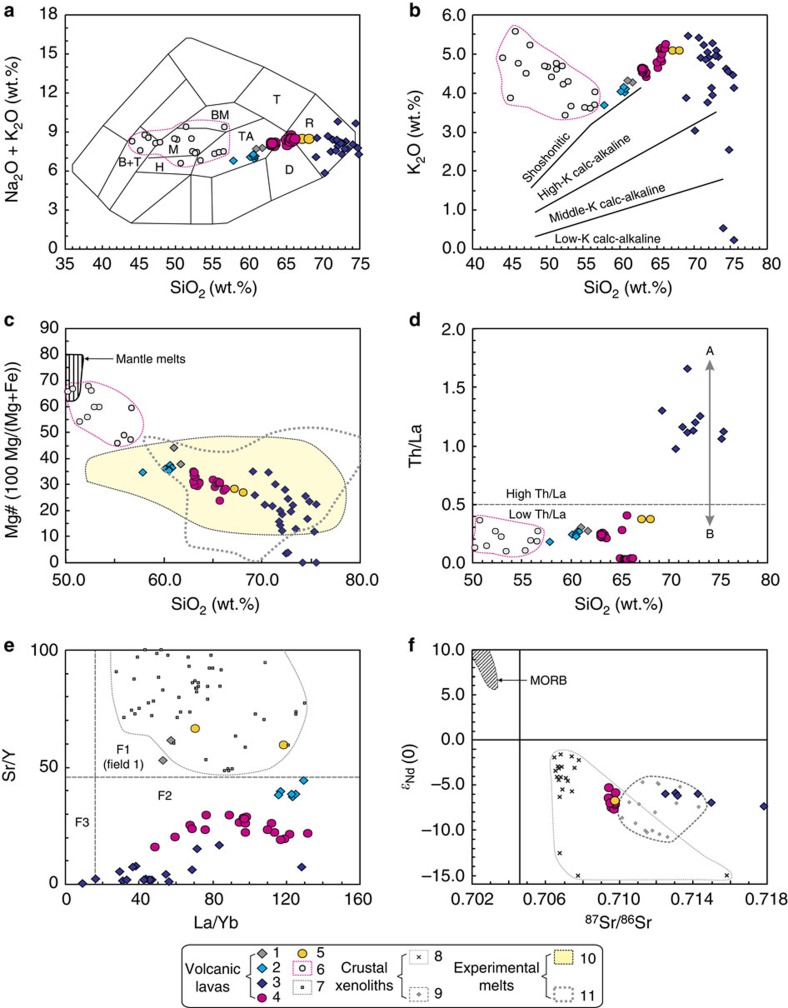
Geochemical diagrams for Late Cenozoic lavas from central and northern Tibet. Units 1–5 are the Pliocene-Quaternary (4.7–0.3 Myr ago) felsic volcanic lavas of central and northern Tibet, with the same key as for Fig. 4. The geochemical data are from Supplementary Data set 1. 6, Miocene (18–13 Myr ago) and Pliocene-Quaternary (5.0–0.07 Myr ago) potassic–ultrapotassic mafic rocks in northern Tibet^5^,^31^; 7, the central and northern Tibetan 47–15 Myr ago adakitic rocks^32^,^33^; 8, granulite xenoliths from 3.8 Myr ago lavas of the Wulanwulahu area of the Qiangtang Block (Supplementary Table 1); 9, garnet-bearing mafic granulite and amphibolite xenoliths from 28 Myr ago intrusive rocks in the Hoh Xil area of Songpan-Ganzi, northern Tibet (Supplementary Table 1). Experimental melts: 10, metabasaltic and eclogite experimental melts (1–4.0 GPa) (after Wang *et al.*^67^); 11, metabasaltic experimental melts (0.1–0.8 GPa)^40^,^43^,^68^. (**a**) SiO_2_ versus K_2_O+Na_2_O (after Cox *et al.*^69^). Rock types: B+T, basanite and tephrite; BM, benmorite; D, dacite; H, hawaiite; M, mugearite; R, rhyolite; T, trachyte; TA, trachyandesite. (**b**) SiO_2_ versus K_2_O. The Pliocene-Quaternary (4.7–0.3 Myr ago) felsic lavas plot on a different trend from northern Tibetan potassic–ultrapotassic mafic rocks. (**c**) SiO_2_ versus Mg#. The field for mantle melts is after Wang *et al.*^67^. The Pliocene-Quaternary (4.7–0.3 Myr ago) felsic lava samples have Mg# lower than the northern Tibetan potassic–ultrapotassic mafic rocks but similar to experimental melts. (**d**) SiO_2_ versus Th/La. High-Th/La ratios in magmatic rocks indicate relatively large contributions from sedimentary source rocks^32^,^36^,^39^. ‘A' indicates partial melting of sediment-dominated crustal sources and ‘B' reflects partial melting of more mafic crustal sources. (**e**) La/Yb versus Sr/Y diagram. This diagram indicates the effects of residual garnet (Grt) and plagioclase (Pl) during partial melting (for discussion see the text). F1 (Field 1), adakitic melts derived from eclogitic rocks in the stability field of Grt with little or no Pl; F2 (Field 2), crustal melts in the stability field of Pl and Grt; F3 (Field 3), crustal melts in the stability field of Pl with little or no Grt. (**f**) ^87^Sr/^86^Sr versus *ɛ*_Nd(0)_ diagram. The field for MORB (middle oceanic ridge basalt) is after Wang *et al.*^67^.

The Pliocene-Quaternary felsic lavas from central and northern Tibet plot in the fields of trachyandesite, dacite and rhyolite in SiO_2_ versus (K_2_O+Na_2_O) diagrams (Fig. 5a). Except for some more evolved rocks that plot in middle- and low-K calc-alkaline fields, the Pliocene-Quaternary felsic lavas in central and northern Tibet are predominantly shoshonitic/high-K calc-alkaline (Fig. 5b). They have high SiO_2_ (58–76 wt.%), low MgO (2.67–0.0 wt.%) and Mg# (<45) (Fig. 5c), and variable K_2_O/Na_2_O (0.54–1.90), except for two high SiO_2_ (72–73 wt.%) samples with high K_2_O/Na_2_O (2.64–10.4) (Supplementary Data set 1). They are enriched in light rare earth elements (LREE) and depleted in heavy rare earth elements (HREE) with negative Nb and positive Pb anomalies (Supplementary Fig. 2). Some samples from the Henglianghu and Zhaixinshan areas have positive or negligible Eu and negligible or slight negative Sr anomalies; all other samples exhibit slight to more marked negative Eu and Sr anomalies (Supplementary Fig. 2; Supplementary Data set 1). The Qiangtang lavas and the Songpan-Ganzi and Central Kunlun trachyandesites have low-Th/La (0.37–0.03) ratios, whereas the Songpan-Ganzi and Central Kunlun rhyolites have distinctive high Th/La (1.66–0.97) ratios (Supplementary Data set 1; Fig. 5d). The Hudongliang and Zhaixinshan lavas tend to have high-Sr/Y and -La/Yb ratios with low-Y and -Yb contents similar to Eocene-Miocene adakitic rocks in central and northern Tibet^32^,^33^, whereas most of the lava samples from other areas have low-Sr/Y and high-La/Yb ratios and variable Y and Yb contents (Fig. 5e; Supplementary Data set 1). The central-northern Qiangtang lavas have isotopically enriched Nd-Sr isotopic compositions (*ɛ*_Nd(0)_=−5.3 to −7.6, ^87^Sr/^86^Sr=0.7094 to 0.7098) (Fig. 5f), within the field for those of garnet-bearing mafic granulite and amphibolite xenoliths from 28 Myr ago intrusive rocks in the Hoh Xil area of the Songpan-Ganzi Block, northern Tibet (Figs 1a and 5f) (Supplementary Table 2). The Songpan-Ganzi and Central Kunlun rhyolitic lavas also have restricted enriched Nd (*ɛ*_Nd(0)_=−5.8 to −8.6) and relatively variable Sr (^87^Sr/^86^Sr=0.7081 to 0.7179) isotope compositions (Fig. 5f), in this case similar to those of granulite xenoliths from 3.8 Myr ago lavas in the Wulanwulahu area, central Tibet (Fig. 5f) (Supplementary Table 1).

### Petrogenesis

Turner *et al.*^5^ interpreted 13.3–0.3 Myr ago potassic–ultrapotassic lavas in Tibet to have been generated by partial melting of relatively old enriched mantle, consistent with their high K_2_O (Fig. 5a,b) and light rare-earth-element contents, and enriched Sr-Nd isotope compositions. Hacker *et al.*^23^ pointed out that if the lower crust beneath Tibet is partly metasedimentary, it is likely to have interacted with hot mantle-generated melts. Indications of *in situ* melting of biotite, feldspar, and quartz in the metasedimentary xenoliths, and of resorption of biotite, feldspar and quartz in the volcanic rocks hosting the xenoliths, imply that partial melts of xenolith material or other metasedimentary rocks did contribute to the mantle-derived magmas^23^. The widespread occurrence of fine-grained, undigested xenocrysts suggests that the unusual chemical patterns of some Tibetan lavas might be a mixture of lower Tibetan crustal fragments with mantle-derived melt^23^. However, we suggest that models of partial melting of enriched mantle^5^, and of mixing between crust- and mantle-derived magmas or crustal assimilation of mantle-derived magmas^23^ cannot account for the formation of the Pliocene-Quaternary felsic lavas reported here.

First, the Pliocene-Quaternary felsic lavas reported here are not potassic–ultrapotassic lavas, which typically contain clinopyroxene, only rare biotite and no amphibole^5^,^31^. Potassic rocks should have K_2_O>2 wt.%, K_2_O/Na_2_O=1.0–2.0, MgO>3–4 wt.% and SiO_2_<55–57 wt.%, and ultrapotassic rocks have K_2_O>3 wt.%, K_2_O/Na_2_O>2.0–3.0, MgO>3–4 wt.% and SiO_2_<55–57 wt%^37^. The Pliocene-Quaternary felsic lavas reported here have high SiO_2_ (>58 wt.%), low MgO (<3.0 wt.%) and Mg# (<45), and variable K_2_O/Na_2_O (0.54–1.90) (Supplementary Data set 1), inconsistent with the definition of potassic–ultrapotassic magmatic rocks.

Second, the high SiO_2_ and low MgO and Mg# values (Supplementary Data set 1; Fig. 5c) of the Pliocene-Quaternary felsic lavas, and the absence of contemporary mantle-derived potassic–ultrapotassic magmatic rocks in the area (Fig. 1b), suggest that these lavas were not directly derived from the upper mantle and do not reflect crustal assimilation of mantle-derived magmas. Miocene (18–13 Myr ago) potassic–ultrapotassic mafic rocks are exposed in the Songpan-Ganzi and the Central Kunlun Blocks (Fig. 1b), and some Pliocene-Quaternary (5.0–0.07 Myr ago) potassic–ultrapotassic mafic rocks occur in western Kunlun area^5^,^30^,^31^, highlighting the absence of contemporary mantle-derived potassic–ultrapotassic magmatic rocks in the studied area. The lavas in the Dongyue Lake and Wulanwula areas contain minor small fine-grained granulite xenoliths or xenocrysts^23^, indicating that they might be mixtures of lower Tibetan crustal fragments with mantle-derived melt^23^. However, these small fine-grained granulite xenoliths or xenocrysts may also be residues after high-temperature crustal melting^38^ although this needs further work. The groundmass glass of the Dongyue dacites has 70–75 wt.% SiO_2_ and <0.5 wt.% FeO+MgO^23^, and as a whole the lavas in the Dongyue Lake and Wulanwula areas exhibit dacitic or trachyandesitic compositions (Fig. 5a), which are markedly different from the mantle-derived northern Tibetan potassic–ultrapotassic lavas with lower SiO_2_ and higher Mg# values (Fig. 5c).

Third, the low-Mg# values, whole-rock Nd-Sr isotope and trace element compositions of the Pliocene-Quaternary felsic lavas support a crustal melting model. Their low-Mg# values are similar to those of experimental melts of metabasaltic rocks or eclogites (Fig. 5c). Their whole-rock Nd-Sr isotope ratios are similar to those of other crustal samples (granulite and amphibolite xenoliths from Cenozoic magmatic rocks) in the area (Fig. 5f) and the high-Th/La ratios of the rhyolites indicate a significant sediment contribution from their source rocks^32^,^36^,^39^. The central-northern Qiangtang lavas have Sr-Nd isotope compositions similar to garnet-bearing mafic granulite and amphibolite xenoliths from ∼28 Myr ago intrusive rocks in the Hoh Xil area of the Songpan-Ganzi Block (Fig. 5f), and low-Th/La ratios (Fig. 5d), suggesting crustal source rocks with a greater contribution of mafic material^36^.

The mineral assemblages in the crustal source rocks of the Pliocene-Quaternary felsic rocks can be further constrained by their geochemical characteristics. Given that plagioclase is strongly enriched in Sr and Eu, and garnet is strongly depleted in LREEs and enriched in HREEs and Y, the distinct negative Sr and Eu anomalies, and the high-La/Yb and low-Sr/Y ratios of most of the Pliocene-Quaternary lavas (except for a few adakitic rocks) (Fig. 5e; Supplementary Fig. 2; Supplementary Data set 1), reflect the presence of residual plagioclase and garnet in their sources^36^,^40^,^41^. Moreover, given that rutile is strongly enriched in Nb, negative Nb anomalies in felsic adakitic rocks commonly indicate crustal melts derived from eclogitic rocks in the stability field of rutile^42^. Thus, the samples from the Henglianghu and Zhaixinshan areas with adakitic characteristics (small variable Eu and Sr and negative Nb anomalies, and high-La/Yb and -Sr/Y ratios and low Yb and Y) (Fig. 5e; Supplementary Fig. 2; Supplementary Data set 1), may contain a greater contribution from eclogitic rocks in their source regions (Fig. 5e), that is, residual garnet+rutile and little or no plagioclase^40^,^41^,^42^,^43^,^44^.

### Temperatures and pressures

Zircon saturation temperatures (*T*_zr_) have been calculated for Pliocene-Quaternary intermediate-acid magmatic rocks in central and northern Tibet using the zircon saturation thermometer of Watson and Harrison^45^ (Supplementary Data set 1). The lavas in the central-northern Qiangtang area and the Jindinghan trachyandesites in northern Tibet have higher zircon saturation temperatures (*T*_zr_=896–983 °C) than the rhyolite and adakitic trachyandesite (for example, Zhaixinshan) lavas in the Songpan-Ganzi and Central Kunlun Blocks (*T*_zr_=700–844 °C)^45^ (Supplementary Data set 1; Fig. 4b). The low-*T*_zr_ (700–844 °C) Pliocene-Quaternary lavas from the Songpan-Ganzi-Central Kunlun area appear to have been derived by partial melting of sediment-dominated crustal rocks (they have elevated Th/La, Fig. 5d) in some cases with contributions from eclogitic source rocks (for example, adakitic trachyandesites with low Th/La but high La/Yb and Sr/Y, Fig. 5d,e). In contrast, the high-*T*_zr_ (896–983 °C) lavas from the central-northern Qiangtang and Songpan-Ganzi-central Kunlun areas are thought to reflect higher temperature melting of more mafic crustal source rocks (Figs 4b and 5d). The Henglianghu adakitic rocks from the northern Qiangtang Block have the highest *T*_zr_ (968–983 °C) (Supplementary Data set 1; Fig. 4b), consistent with partial melting of eclogite-facies crustal rocks (Fig. 5d) at>960 °C.

**Figure 6:**
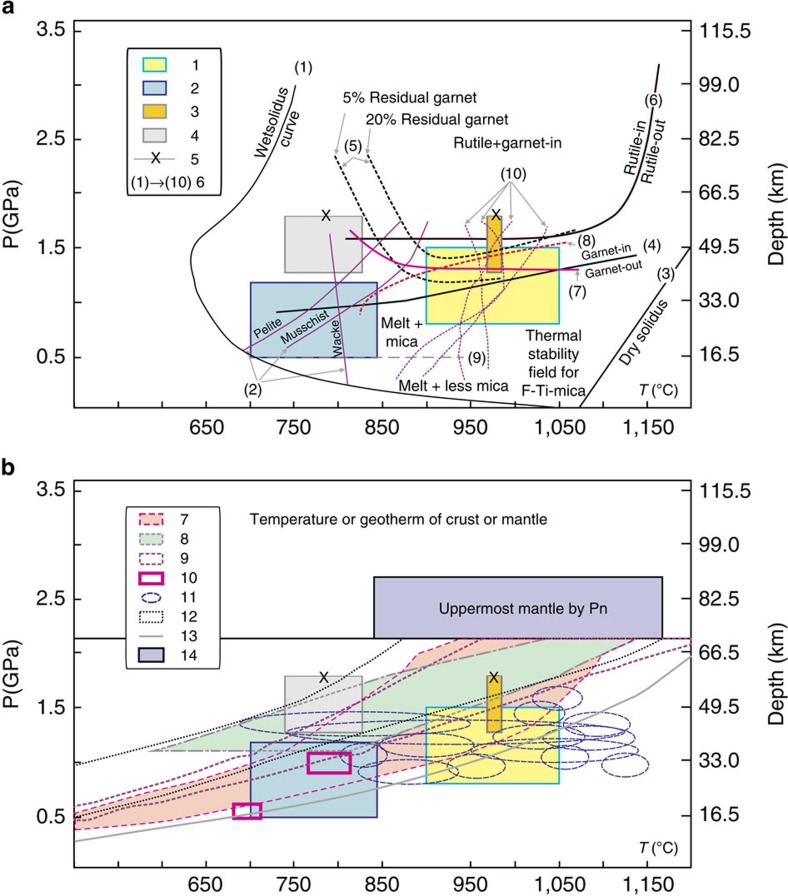
Crustal thermal state for central and northern Tibet. (**a**) Pressure–temperature conditions for partial melting of crustal rocks. 1–4, pressure and temperature conditions for magma generation (Supplementary Data set 1): (1) Quaternary (1.08–0.3 Myr ago) non-adakitic trachyandesites of the Songpan-Ganzi and Central Kunlun Blocks and Pliocene-Quaternary (4.7–2.3 Myr ago) non-adakitic felsic volcanic lavas from the central-northern Qiangtang Block; (2) Pliocene-Quaternary (4.0–1.5 Myr ago) rhyolites in the Songpan-Ganzi and Central Kunlun Blocks; (3) Pliocene (3.2–2.5 Myr ago) adakitic rhyolites in the Henglianghu area of the central-northern Qiang Block; (4) Pliocene (2.97 Myr ago) adakitic trachyandesites in the Zhaixinshan area of the Central Kunlun Block. (5) Adakitic magmas generated in the pressure range >1.2–1.5 GPa. (6) curves or lines for melting or mineral stability: (1) H_2_O-saturated or wet solidus curve of crustal rocks^42^,^48^,^70^; (2) dehydration solidi curve for crustal rocks^17^,^47^; (3) dry solidus curve^70^; (4) garnet-in/out curve during dehydration melting of metabasalts^40^,^42^,^43^,^44^,^47^,^48^; (5) garnet proportion contours (5 and 20 wt.%) in the residue of metabasalt melting^44^; (6) rutile-in/out curve^42^; (7) plagioclase-in (beneath curve)/out (above curve) curve during dehydration melting^43^,^70^; (8) orthopyroxene-in (beneath curve)/out (above curve) curve during dehydration melting^70^; (9) the boundary line for garnet-bearing (above line) and cordierite-rich (beneath line) melts during dehydration melting of metasedimentary rocks^36^; (10) upper thermal stability of F-free mica in pelite, wacke, mica schist and tonalite^17^,^47^,^48^. The fields for melt+mica, melt+minor mica and thermal stability field for F-Ti-mica are after Hacker *et al.*^17^. (**b**) Constraints on the variations in pressure and temperature (thermal gradient) in the central and northern Tibetan crust. (7) Temperature in central Tibetan crust^17^; (8) temperature in the Qiangtang crust^53^; (9) temperature in the Qiangtang crust^55^; (10) temperature for α-β transition^52^; (11) xenolith temperatures for the Qiangtang crust^23^; (12) geotherm of the Northern Qiangtang-South Qaidam crust^54^; (13) geothem of the Northern Songpan crust^35^; (14) temperature of uppermost mantle by Pn^51^. These data are from geophysical^17^,^52^,^53^,^54^, crustal rock or granulite xenolith^17^,^23^,^35^, and geophysical-petrological modeling studies^55^.

Pressure–temperature conditions for partial melting of crustal rocks based on experimental data are summarized in Fig. 6a. They indicate that garnet forms at pressures of 0.5–0.6 GPa and temperatures of 750–900 °C during partial melting of metasedimentary rocks^36^,^46^,^47^, and that the lower limit of garnet stability is 0.5 GPa (Line 9 in Fig. 6a). Plagioclase is a common residual mineral during partial melting of metasedimentary and igneous rocks (such as tonalites and basalts), but it disappears at pressures >1.2–1.5 GPa^40^,^42^,^43^,^48^ (Fig. 6a), and the lower limit of rutile stability is typically 1.5 GPa^42^ (Fig. 6a). Given the evidence for residual garnet and plagioclase and the zircon saturation temperature data, we interpret the Songpan-Ganzi-central Kunlun rhyolites to have been generated by dehydration partial melting of metasedimentary source rocks in the temperature and pressure ranges of 700–844 °C and 0.5–1.2 GPa (Fig. 6a) (corresponding to depths of 15–40 km^36^ (Supplementary Data set 1; Fig. 4c)). The more adakitic Zhaixinshan magmas were derived by partial melting of eclogitic rocks with residual garnet+rutile and little or no plagioclase at temperatures and pressures of 741–825 °C and >1.2–1.5 GPa^32^,^33^ (Fig. 6a) (corresponding to depths of 40–50 km (Supplementary Data set 1; Figs 4c and 5e)).

The combination of residual garnet and plagioclase in the source, and the high-*T*_zr_ of lavas, except for the Henglianghu adakitic rocks from central-northern Qiangtang and Songpan-Ganzi-central Kunlun (Supplementary Data set 1; Fig. 4b), suggests that they were generated by fluid-absent melting of granulite-facies crustal rocks at >900 °C (Fig. 6a). This is consistent with the occurrence of titaniferous magnetite and ilmenite and F-Ti-rich mica in the Dongyue Lake dacites (Supplementary Table 3), suggesting that their magmas were generated in H_2_O-poor and high-temperature (>950 °C) conditions given the stability of titaniferous magnetite in extremely H_2_O-poor^49^ and F-Ti-rich mica in high-temperature^17^,^23^ (Fig. 6a) environments. Monazites from the granulite xenoliths in the Dongyue Lake dacites have ^208^Pb/^232^Th ages ranging from 16 to 2.9 Myr ago^23^, possibly indicating young granulite-facies metamorphic and/or melting events before entrainment in the dacitic lavas. Pressure–temperature calculations suggest that the Dongyue Lake granulite xenoliths formed at pressures of 0.8–1.5 GPa and temperatures of 800–1,100 °C^23^ (Fig. 6b), consistent with fluid-absent melting of metasedimentary and igneous rocks (0.7–1.5 GPa and 900–1,050 °C)^36^,^40^,^43^,^46^,^47^,^48^. In summary, most of the high-T_zr_ lavas from the central-northern Qiangtang and Songpan-Ganzi-Central Kunlun areas are attributed to fluid-absent melting of granulite-facies rocks at temperatures and pressures of 900–1,050 °C and 0.8–1.5 GPa (Fig. 6a) (corresponding to crustal depths of 26–50 km (Supplementary Data set 1; Fig. 4c). The Henglianghu adakitic rocks in contrast appear to have been derived by partial melting of eclogitic rocks with residual garnet+rutile and little or no plagioclase at temperatures and pressures of 968–983 °C and >1.2–1.5 GPa (Fig. 6a) (corresponding to crustal depths of 40–50 km (Supplementary Data set 1; Fig. 4c)).

## Discussion

There is some west–east variation in the thermal state of southern Tibet^50^. However, granulite xenolith^17^,^23^ and most geophysical^1^,^17^,^26^,^28^,^51^,^52^,^53^,^54^,^55^ data show that, compared with southern Tibet, there is a relatively hot mid-to-lower crust (up to 1,150 °C at the Moho) and lithospheric mantle lid (more than 1,200 °C) beneath central and northern Tibet. The estimated geotherms for central and northern Tibetan crust from geophysical, crustal xenolith and geophysical/petrological modelling studies^17^,^23^,^35^,^52^,^53^,^54^,^55^ are shown in Fig. 6b. Moreover, the estimated temperatures and pressures for the generation of the Pliocene-Quaternary felsic magmas are consistent with the present crustal geotherms for central and northern Tibet (Fig. 6b), indicating that high temperatures in the mid-lower crust of central and northern Tibet were responsible for the fluid-absent partial melting. These elevated temperatures in central and northern Tibet have been attributed to upwelling asthenosphere in response to lower lithosphere delamination^4^,^5^, mantle counterflow coupled with the northward downwelling of the Indian mantle lithosphere or the southward downwelling of the Asian mantle lithosphere^1^,^28^, and to squeezing between the northward advancing Indian and resisting Qaidam and Tarim lithospheres^30^,^50^. In all these models, heat conducted from the underlying hot lithospheric mantle heated the mid-to-lower crust, which melted to form felsic magmas in central and northern Tibet (Fig. 2). Radioactive isotopes may possibly have been introduced to the mid-to-lower crust beneath central and northern Tibet in sediments during India–Eurasia convergence or pre-Cenozoic subduction^2^,^6^,^23^, and they would have provided an additional heat source for crustal melting and the generation of felsic magmas^56^,^57^.

The presence of crust-derived Pliocene-Quaternary (4.7–0.3 Myr ago) felsic rocks in central and northern Tibet (Figs 1b, 2 and 4) provides new evidence as to the nature of the LV–HCZs within the crust beneath Tibet. The LV–HCZs occur at depths of 15–50 km, similar to the depths at which the Pliocene-Quaternary crust-derived magmas were generated (Figs 1b, 2 and 4), and the felsic magmas are restricted to the areas of the largest negative values (−12 to −6%) for the amplitude of the Vs perturbation associated with the LVZ (Fig. 1a) and the lowest shear wave speeds (*V*_s_<3.35 or 3.25 km s^−1^)^17^,^22^ (Fig. 4c). The MT data also indicate that the lowest resistivities occur in the middle crust beneath the northern Qiangtang Block^14^,^15^,^21^,^26^,^27^, where the Pliocene-Quaternary crust-derived felsic rocks occur. These results are consistent with experimental results and model calculations of the seismic properties of partially molten rocks that strongly suggest that the electrical and seismic anomalies measured beneath the Tibetan Plateau are best explained by the presence of partially molten rocks^17^,^26^,^58^,^59^.

In this study, simple batch melting models, constrained by estimates of Rb/Sr in the source from Nd and Sr isotopes^60^ and REE contents of the Pliocene-Quaternary lavas of central and northern Tibet, indicate that the crustal melts from central and northern Tibet reflect 8–22% partial melts (Supplementary Tables 4–11). Such degrees of partial melting are those present at the time the volcanic magmas were generated, and the youngest of those is 0.3 Myr ago old. In contrast, the amounts of melt at depth at the present day is constrained geophysically and, based on MT data from Tibet, the estimates for the amounts of melt required to explain the HCZs in Tibet range from 5 to 23%^20^,^25^,^26^. Melting and numerical experiments also suggest that the melt fractions required to explain the HCZs in Tibet were 8–23%(refs 58, 59).

While such estimates of 8–23% are similar to those from the geochemical data in this study and the MT data in Tibet, both are higher than the melt fractions (∼1–5%) suggested by a number of seismic studies^17^,^61^. In practice, melt fractions estimated from seismic and MT data are not always in agreement, and seismic studies in Tibet have generally given lower estimates of melt fractions compared with those derived from MT studies^26^. Le Pape *et al.*^26^ provided a detailed discussion of what factors might affect these values and how they might be reconciled. Seismic velocity varies by a factor of <3 for the melt range discussed, whereas the resistivity can vary by 3 orders of magnitude^26^,^62^. Thus, electrical resistivity is more sensitive to the size of the melt fractions.

The inferred melt fraction estimates from the resistivity models are also consistent with values predicted from fluid-absent melting petrological models at the observed P/T conditions and for similar compositions^26^. For instance, at 1 GPa and 900 °C in the presence of 0.6 wt.% H_2_O (consistent with the mineralogy of Qiangtang defined by Hacker *et al.*^14^), Le Pape *et al.*^26^ predicted melt fractions of ∼10–20% for dehydration melting of pelites and quartzofelspathic rocks, intermediate rocks and mafic rocks. These are in good agreement with those (8–22% and 5–23%) calculated in this study, and estimated from MT and numerical experiment data^20^,^25^,^26^,^58^,^59^. In Tibet, the lower resistivities in the middle crust beneath the northern Qiangtang Block, indicating where the higher melt fractions occur^27^, are in the area where the Pliocene-Quaternary felsic lavas outcrop (Figs 1, 2 and 4c).

Taken together the geochemistry and MT data indicate that ∼10–20% partial melt is present today in the high-conductivity zones (LV–HCZs), and that similar degrees of melting were required to generate the volcanic rocks in the period 4.7–0.3 Myr ago. This indicates that conditions in the mid-to-lower crust would have facilitated tectonic movements in this region for at least the last 5 Myr ago.

In summary, three main mechanisms (intracontinental subduction, lithosphere thinning and crustal flow) have been proposed to account for the crustal thickening and high topography in Tibet. The first two emphasize changes in lithospheric mantle structure^2^,^4^,^5^,^6^ (Fig. 2), and they may have occurred at relatively early stages in the development of the Tibetan Plateau^1^,^36^, or at the margin of the Tibetan Plateau (for example, crustal brittle thickening in northern part of western Kunlun^63^ or Qilian areas^2^,^6^ (Fig. 1a)). In contrast, crustal flow involves a decoupling of movement in the upper crust from those in the high-temperature mid-to-lower crust, probably during the relatively late heating and eventual melting of the crust in response to the continuous convergence between the Indian and Eurasian plates^1^,^36^ (Fig. 2).

Crustal flow requires a layer with a viscosity less than that of the adjacent rocks and an effective viscosity below an absolute threshold that is dependent on layer thickness^7^,^11^,^20^. In a re-evaluation of the experimental data, Rosenberg and Handy^64^ suggested a melt fraction of ∼7 vol% as the ‘melt connectivity transition', marking the increase of melt-interconnectivity that causes the dramatic strength drop. The mechanical response of crust containing a layer with 8% melt may be very different from one that contains only 2% melt, but it will not differ markedly from that of crust containing a layer with 50% melt, despite their different microstructures and compositions^65^. If only 1–2% and 3–5% melts now occur in the crust of the Qiangtang and Songpan-Ganzi Blocks, as suggested by the seismic data^17^,^61^, the strength of the mid-lower crust would not have been significantly changed^64^. Alternatively, if there are ∼10–20% melts in the mid-lower crust of the Qiangtang and Songpan-Ganzi Blocks, as suggested by MT and the new Pliocene-Quaternary felsic lava data, the strength of the mid-lower crust beneath central and northern Tibet will have been markedly changed^59^,^64^. This in turn would facilitate northward and eastward flow of melt-weakened mid-lower crust of central and northern Tibet^12^,^25^,^26^,^36^,^66^.

Crustal melt-enhanced ductile flow in the high-temperature, partially molten, mid-to-lower crust makes it easier to maintain a uniform elevation in the Tibetan Plateau^1^,^19^,^36^ and it accounts for the present expansion and frequent earthquakes along its northern and eastern margins^3^,^12^,^25^,^26^,^36^,^66^. Similar examples of crustal melt-enhanced ductile flow are likely to have occurred elsewhere (for example, the modern Andean and Anatolian plateaus), shaping the deep structure of the Earth's continental crust and restraining the thickness and elevation of mountain belts^19^,^36^.

## Methods

### Zircon U-Pb age analyses

Zircon U-Pb analyses for sample 5133-1 and samples 11WL59-2 and 11WL60-3 were conducted using the Cameca IMS-1280 SIMS (CASIMS) at the Institute of Geology and Geophysics, Chinese Academy of Sciences (IGGCAS) and the State Key Laboratory of Isotope Geochemistry, Guangzhou Institute of Geochemistry, Chinese Academy of Sciences (SKLaBIG GIGCAS), respectively. The O_2_^−^ primary ion beam with an intensity of *ca.* 10 nA was accelerated at −13 kV. The ellipsoidal spot is about 20 × 30 μm in size. The aperture illumination mode (Kohler illumination) was used with a 200 μm primary beam mass filter aperture to produce even sputtering over the entire analysed area. In the secondary ion beam optics, a 60 eV energy window was used, together with a mass resolution of ca. 5,400. Rectangular lenses were activated in the secondary ion optics to increase the transmission at high-mass resolution. A single electron multiplier was used on ion-counting mode to measure secondary ion beam intensities by peak jumping sequence: 196 (^90^Zr_2_^16^O, matrix reference), 200 (^92^Zr_2_^16^O), 200.5 (background), 203.81 (^94^Zr_2_^16^O, for mass calibration), 203.97 (Pb), 206 (Pb), 207 (Pb), 208 (Pb), 209 (^177^Hf^16^O_2_), 238 (U), 248 (^232^Th^16^O), 270 (^238^U^16^O_2_) and 270.1 (reference mass), 1.04 s, 0.56 s, 4.16 s, 0.56 s, 6.24 s, 4.16 s, 6.24 s, 2.08 s, 1.04 s, 2.08 s, 2.08 s, 2.08 s and 0.24 s, respectively. Each measurement consisted of seven cycles, and the total analytical time is *ca.* 12 min. Calibration of Pb/U ratios is relative to the zircon standard TEMORA 2 (417 Myr ago) based on an observed linear relationship between ln(^206^Pb/^238^U) and ln(^238^U^16^O_2_/^238^U). U and Th concentrations of unknowns were determined relative to the standard zircon 91500 (1,065 Myr ago) with Th and U concentrations of *ca.* 29 p.p.m. and 81 p.p.m., respectively. Measured compositions were corrected for common Pb using non-radiogenic ^204^Pb. Uncertainties on individual analyses are reported at 1σ level; mean ages for pooled U-Pb analyses are quoted at 95% confidence.

### Geochemical and mineral composition analyses

Whole-rock geochemical and mineral composition analyses were carried on at the SKLaBIG GIGCAS. Major elements were measured on individual minerals using a JEOL JXA-8100 Superprobe with an accelerating potential of 15 kV and sample current of 20 nA. Whole-rock major element oxides (wt.%) for whole-rock powders were determined using a Varian Vista PRO ICP-AES using wavelength X-ray fluorescence spectrometry with analytical errors better than 2%. Whole-rock trace elements, including the REEs, were analysed using a Perkin-Elmer ELAN 6000 inductively-coupled plasma source mass spectrometer (ICP-MS). Analytical precision for most elements is better than 3%. Whole-rock Sr and Nd isotopic compositions of selected samples were determined using a Micromass Isoprobe multi-collector mass spectrometer (MC-ICP-MS). The ^87^Sr/^86^Sr ratio of the NBS987 standard and ^143^Nd/^144^Nd ratio of the Shin Etsu JNdi-1 standard measured were 0.710288±28 (2σ) and 0.512109±12 (2σ), respectively. All measured ^143^Nd/^144^Nd and ^86^Sr/^88^Sr ratios are fractionation corrected to ^146^Nd/^144^Nd=0.7219 and ^86^Sr/^88^Sr=0.1194, respectively.

### Partial melting calculations

The calculated degrees of partial melting for the crust-derived magmas are based on simple batch melting models. These are constrained by comparing estimates of Rb/Sr in the source from combined Nd and Sr isotope ratios with the Rb/Sr ratios of the rocks analysed, and the REE contents of the felsic magmatic lavas together with appropriate distribution coefficients for the different minerals. The detailed methods and equations are presented in Supplementary Methods.

## Additional information

**How to cite this article:** Wang, Q. *et al.* Pliocene-quaternary crustal melting in central and northern Tibet and insights into crustal flow. *Nat. Commun.* 7:11888 doi: 10.1038/ncomms11888 (2016).

## Supplementary Material

Supplementary Information Supplementary Figures 1-2, Supplementary Tables 1-11, Supplementary Methods and Supplementary References.

Supplementary Data 1 Geochemical and Geochronological data for Pliocene-Quaternary felsic volcanic rocks in central and northern Tibetan Plateau.
