# Supplementary material for: Pliocene-Quaternary crustal melting in central and northern Tibet and insights into crustal flow
Source: Nat Commun. 2016 Jun 16;7:11888. doi: 10.1038/ncomms11888 (PMC4912662; doi:10.1038/ncomms11888)
Supplement: Supplementary Information — Supplementary Figures 1-2, Supplementary Tables 1-11, Supplementary Methods and Supplementary References. [file ncomms11888-s1.pdf]

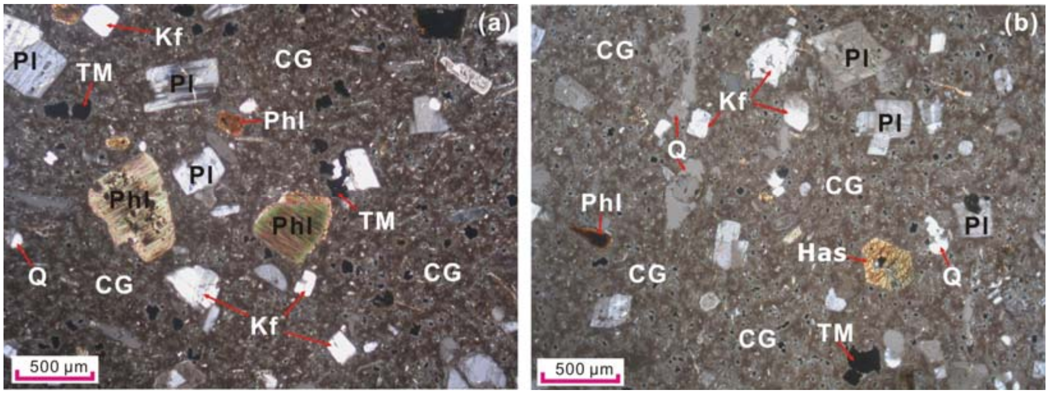

Supplementary Figure 1 The Dongyue Lake dacites with porphyritic textures and cryptocrystalline-glassy groundmass. (**a**) sample 5127-3, crossed polarized light (xpl); (**b**) sample 5124-1, crossed polarized light (xpl). Phenocryst minerals：plagioclase (Pl) + K-feldspar (Kf) + phlogopite (Phl) + quartz (Q) + titaniferous magnetite (TM) + hastingsite (Has); Groundmass：microcrystalline K-feldspar (Kf) and titaniferous magnetite (TM); cryptocrystalline-glassy groundmass (CG).

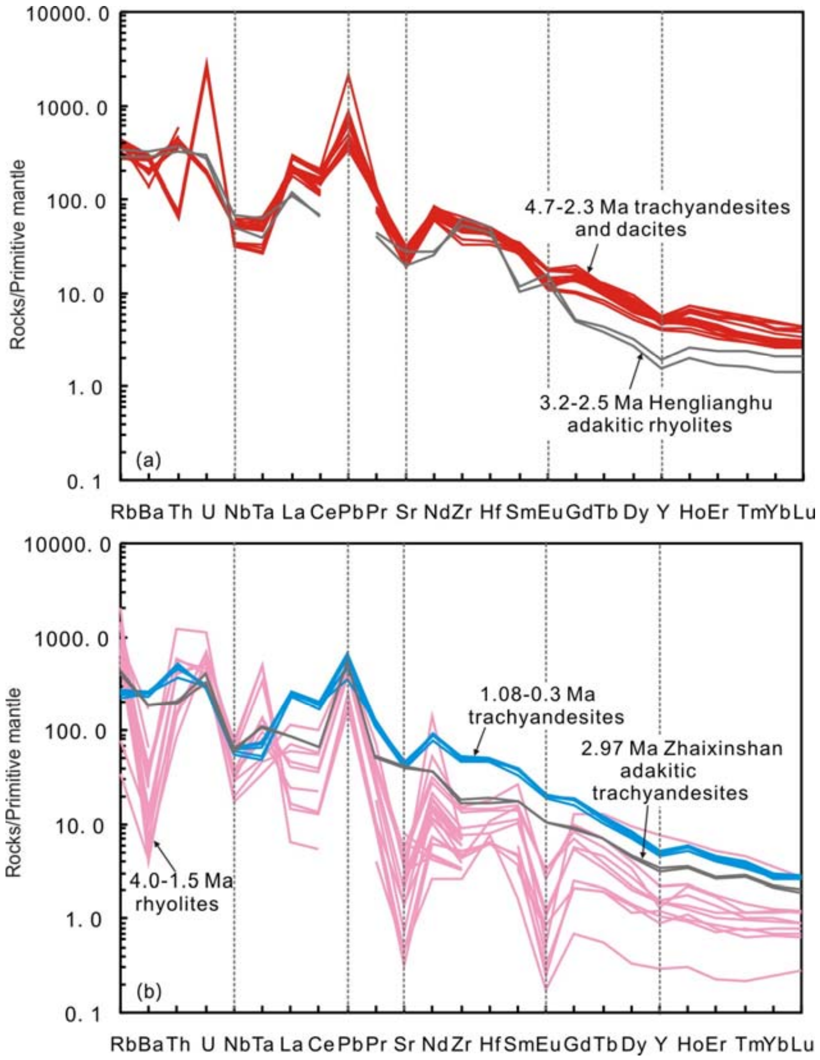

Supplementary Figure 2 Primitive mantle-normalized multi-element patterns for lavas. Primitive mantle values are from Sun & McDonough[13]. (**a**) 4.7−2.3 Ma felsic lavas from central-northern Qiangtang, central Tibet. (**b**) 4.0−0.3 Ma felsic lavas from Songpan-Ganzi and Central Kunlun areas in northern Tibet. Given that plagioclase (Pl) is strongly enriched in Sr and Eu, and garnet (Grt) is strongly enriched in heavy rare earth elements (HREEs) and Y, positive or negligible Sr and Eu anomalies together with HREE and Y depletion in felsic adakitic rocks commonly indicates crustal melts derived from eclogitic rocks in the stability field of garnet with little or no plagioclase. Negative Sr and Eu anomalies with HREE and Y depletion in felsic rocks commonly indicates crustal melts from the stability field of Grt and Pl[14-15]. Given that rutile is strongly enriched in Nb, negative Nb anomalies in felsic adakitic rocks commonly indicates crustal melts derived from eclogitic rocks in the stability field of rutile[16].

Supplementary Table 1 Granulite and amphibolite xenoliths from Cenozoic magmatic rocks in central and northern Tibet

Sample [a, b]	11WL59-1-2	11WL59-1-4	11WL59-1-5	11WL59-1-6	11WL59-1-7	11WL59-1-8
rocks	FG	FG	FG	MG	MG	MG
location	Wulanwulahu	Wulanwulahu	Wulanwulahu	Wulanwulahu	Wulanwulahu	Wulanwulahu
Block	Qiangtang	Qiangtang	Qiangtang	Qiangtang	Qiangtang	Qiangtang
Ages of host magmatic rocks (Ma)	3.8 ± 0.2	3.8 ± 0.2	3.8 ± 0.2	3.8 ± 0.2	3.8 ± 0.2	3.8 ± 0.2
Sm	0.16	4.81	12.1	9.97	6.56	3.53
Nd	1.63	31.2	80.7	41.3	36.4	20.3
$^{147}Sm/^{144}Nd$	0.0600	0.0939	0.0912	0.147	0.110	0.106
$^{143}Nd/^{144}Nd$	0.512119	0.512278	0.512283	0.512105	0.512198	0.512229
2 SE	0.000007	0.000007	0.000006	0.000007	0.000006	0.000007
eNd(0)	-10.1	-7.0	-6.9	-10.4	-8.6	-8.0
Rb	553	126	37.9	16.5	41.5	135
Sr	246	129	615	296	433	324
$^{87}Rb/^{86}Sr$	6.51	2.85	0.178	0.161	0.277	1.21
$^{87}Sr/^{86}Sr$	0.718840	0.713044	0.710479	0.712157	0.710133	0.711894
2 SE	0.000007	0.000008	0.000007	0.000006	0.000007	0.000005

a) The FG (felsic granulite) and MG (mafic granulite) xenoliths are from the 3.8 Ma volcanic lavas in the Wulanwulahu area, in the Qiangtang Block of central Tibet; b) The amphibolite and garnet (Grt)-bearing M (mafic) xenoliths are from ~ 28 Ma porphyries in the Gongmaorima area of the Hoh Xil district, northern Tibet; c) These samples are from Lai et al.[17].

Supplementary Table 1 (Continued)

Sample [a, b]	11WL60-1	11WL60-2-1	11WL60-5	11WL60-7	11WL60-8	11WL61-2
Rocks	MG	MG	FG	FG	FG	MG
Location	Wulanwulahu	Wulanwulahu	Wulanwulahu	Wulanwulahu	Wulanwulahu	Wulanwulahu
Block	Qiangtang	Qiangtang	Qiangtang	Qiangtang	Qiangtang	Qiangtang
Ages of host magmatic rocks (Ma)	3.8 ± 0.2	3.8 ± 0.2	3.8 ± 0.2	3.8 ± 0.2	3.8 ± 0.2	3.8 ± 0.2
Sm	4.50	9.28	6.09	1.60	6.39	23.0
Nd	25.7	57.4	29.7	10.3	31.4	133
$^{147}Sm/^{144}Nd$	0.106	0.0984	0.125	0.0943	0.124	0.105
$^{143}Nd/^{144}Nd$	0.512085	0.512257	0.512052	0.512102	0.512066	0.512273
2 SE	0.000006	0.000008	0.000007	0.000006	0.000008	0.000006
eNd(0)	-10.8	-7.4	-11.4	-10.5	-11.2	-7.1
Rb	95.2	16.6	221	375	219	26.6
Sr	430	103	76.4	248	82.6	566
$^{87}Rb/^{86}Sr$	0.641	0.470	8.39	4.37	7.70	0.136
$^{87}Sr/^{86}Sr$	0.712551	0.714281	0.731107	0.718600	0.729577	0.711041
2 SE	0.000008	0.000007	0.000005	0.000005	0.000006	0.000007

Supplementary Table 1 (Continued)

Sample [a, b]	LBT5 [c]	LBT18 [c]	LBT20 [c]	LBT41 [c]	LBT42 [c]	LBT46 [c]
Rocks	MG	MG	MG	MG	MG	MG
Location	Wulanwulahu	Wulanwulahu	Wulanwulahu	Wulanwulahu	Wulanwulahu	Wulanwulahu
Block	Qiangtang	Qiangtang	Qiangtang	Qiangtang	Qiangtang	Qiangtang
Ages of host magmatic rocks (Ma)	3.8 ± 0.2	3.8 ± 0.2	3.8 ± 0.2	3.8 ± 0.2	3.8 ± 0.2	3.8 ± 0.2
Sm	7.13	7.34	8.00	17.7	5.25	14.1
Nd	42.4	41.9	50.0	124	34.5	90.7
$^{147}Sm/^{144}Nd$	0.102	0.106	0.0967	0.0864	0.0920	0.0943
$^{143}Nd/^{144}Nd$	0.512123	0.512112	0.512372	0.512123	0.512165	0.512397
2 SE	0.000005	0.000004	0.000004	0.000005	0.000004	0.000011
eNd(0)	-10.0	-10.3	-5.2	-10.0	-9.2	-4.7
Rb	75.9	61.9	57.8	100	48.5	65.9
Sr	427	450	422	309	492	304
$^{87}Rb/^{86}Sr$	0.515	0.398	0.397	0.936	0.286	0.628
$^{87}Sr/^{86}Sr$	0.711736	0.711470	0.713241	0.711382	0.710812	0.711227
2 SE	0.000007	0.000006	0.000007	0.000017	0.000008	0.000010

Supplementary Table 1 (Continued)

Sample [a, b]	LBT17 [c]	LBT36 [c]	LBT54 [c]	11GM01-1	11GM01-2	11GM01-3
Rocks	FG	FG	FG	Grt-bearing M	Grt-bearing M	Grt-bearing M
Location	Wulanwulahu	Wulanwulahu	Wulanwulahu	Gongmaorima	Gongmaorima	Gongmaorima
Block	Qiangtang	Qiangtang	Qiangtang	Songpan-Ganzi	Songpan-Ganzi	Songpan-Ganzi
Ages of host magmatic rocks (Ma)	3.8 ± 0.2	3.8 ± 0.2	3.8 ± 0.2	27.6 ± 0.5	27.6 ± 0.5	27.6 ± 0.5
Sm	4.32	4.78	2.19	5.95	1.36	2.76
Nd	23.2	25.1	8.88	39.0	4.51	10.5
$^{147}Sm/^{144}Nd$	0.112	0.115	0.149	0.0928	0.183	0.160
$^{143}Nd/^{144}Nd$	0.512388	0.512395	0.51223	0.511845	0.512538	0.512356
2 SE	0.000007	0.000005	0.000009	0.000006	0.000007	0.000008
eNd(0)	-4.9	-4.7	-8.0	-15.5	-2.0	-5.5
Rb	190	183	103	312	305	135
Sr	179	189	80.9	599	644	701
$^{87}Rb/^{86}Sr$	3.07	2.79	3.69	1.40	1.27	0.517
$^{87}Sr/^{86}Sr$	0.712087	0.712041	0.729088	0.716347	0.707885	0.708039
2 SE	0.000009	0.000009	0.000009	0.000008	0.000006	0.000006

Supplementary Table 1 (Continued)

Sample [a, b]	11GM01-16	11GM01-17	11GM01-19	11GM01-20	11GM01-21	11GM01-22
Rocks	Grt-bearing M	Grt-bearing M	Grt-bearing M	Grt-bearing M	amphibolite	amphibolite
Location	Gongmaorima	Gongmaorima	Gongmaorima	Gongmaorima	Gongmaorima	Gongmaorima
Block	Songpan-Ganzi	Songpan-Ganzi	Songpan-Ganzi	Songpan-Ganzi	Songpan-Ganzi	Songpan-Ganzi
Ages of host magmatic rocks (Ma)	27.6 ± 0.5	27.6 ± 0.5	27.6 ± 0.5	27.6 ± 0.5	27.6 ± 0.5	27.6 ± 0.5
Sm	4.88	3.62	8.93	2.92	4.09	3.28
Nd	17.3	16.6	60.5	11.2	23.5	17.4
$^{147}Sm/^{144}Nd$	0.172	0.133	0.090	0.159	0.106	0.115
$^{143}Nd/^{144}Nd$	0.512519	0.511982	0.512453	0.512417	0.512404	0.512341
2 SE	0.000006	0.000008	0.000007	0.000007	0.000006	0.000006
eNd(0)	-2.3	-12.8	-3.6	-4.3	-4.6	-5.8
Rb	200	351	270	46.6	226	179
Sr	407	1456	2061	253	1001	857
$^{87}Rb/^{86}Sr$	1.33	0.649	0.353	0.495	0.607	0.563
$^{87}Sr/^{86}Sr$	0.708279	0.706988	0.706806	0.706791	0.707289	0.707075
2 SE	0.000008	0.000007	0.000007	0.000007	0.000007	0.000007

Supplementary Table1 (Continued)

Sample [a, b]	11GM01-24	11GM01-25	11GM01-26	11GM01-27	11GM01-28	11GM01-29
Rocks	amphibolite	amphibolite	amphibolite	amphibolite	amphibolite	amphibolite
Location	Gongmaorima	Gongmaorima	Gongmaorima	Gongmaorima	Gongmaorima	Gongmaorima
Block	Songpan-Ganzi	Songpan-Ganzi	Songpan-Ganzi	Songpan-Ganzi	Songpan-Ganzi	Songpan-Ganzi
Ages of host magmatic rocks (Ma)	27.6 ± 0.5	27.6 ± 0.5	27.6 ± 0.5	27.6 ± 0.5	27.6 ± 0.5	27.6 ± 0.5
Sm	5.63	10.3	13.1	4.26	16.6	4.85
Nd	28.7	52.6	75.4	24.3	94.1	23.1
$^{147}Sm/^{144}Nd$	0.119	0.119	0.106	0.107	0.107	0.128
$^{143}Nd/^{144}Nd$	0.512397	0.512473	0.512522	0.512535	0.512298	0.512465
2 SE	0.000007	0.000006	0.000008	0.000006	0.000006	0.000009
eNd(0)	-4.7	-3.2	-2.3	-2.0	-6.6	-3.4
Rb	47.4	138	107	214	312	146
Sr	549	1079	1095	879	2168	897
$^{87}Rb/^{86}Sr$	0.233	0.343	0.263	0.657	0.387	0.438
$^{87}Sr/^{86}Sr$	0.706793	0.706779	0.706536	0.707013	0.706882	0.707684
2 SE	0.000008	0.000008	0.000008	0.000008	0.000007	0.000006

Supplementary Table 1 (Continued)

Sample [a, b]	11GM01-30	11GM01-38	11GM01-39	11GM01-48	11GM01-65	11GM01-66
Rocks	amphibolite	amphibolite	amphibolite	amphibolite	amphibolite	amphibolite
Location	Gongmaorima	Gongmaorima	Gongmaorima	Gongmaorima	Gongmaorima	Gongmaorima
Block	Songpan-Ganzi	Songpan-Ganzi	Songpan-Ganzi	Songpan-Ganzi	Songpan-Ganzi	Songpan-Ganzi
Ages of host magmatic rocks (Ma)	27.6 ± 0.5	27.6 ± 0.5	27.6 ± 0.5	27.6 ± 0.5	27.6 ± 0.5	27.6 ± 0.5
Sm	2.77	6.91	2.81	0.66	15.8	3.78
Nd	11.2	35.8	13.7	2.65	90.9	19.4
$^{147}Sm/^{144}Nd$	0.150	0.117	0.125	0.150	0.106	0.118
$^{143}Nd/^{144}Nd$	0.512474	0.512448	0.512414	0.512394	0.511848	0.512332
2 SE	0.000006	0.000008	0.000008	0.000007	0.000006	0.000006
eNd(0)	-3.2	-3.7	-4.4	-4.8	-15.4	-6.0
Rb	324	63.2	211	659	475	150
Sr	785	754	1485	1349	1076	768
$^{87}Rb/^{86}Sr$	1.11	0.226	0.382	1.31	1.19	0.526
$^{87}Sr/^{86}Sr$	0.707260	0.706602	0.706947	0.707651	0.708173	0.707614
2 SE	0.000007	0.000006	0.000007	0.000006	0.000006	0.000007

Supplementary Table 2 CASIMS (Cameca IMS–1280) zircon U-Pb results[a)]

Sample spot	U ppm	Th ppm	Pb ppm	Th/U	f_{206}%[b)]	$^{238}U/^{206}Pb$	±σ(%)	$^{207}Pb/^{206}Pb$	±σ(%)	Age[c)] (Ma)	±σ
Sample 5133-1											
5133-1@1	40.3	79.2	0.055	2.0	31.2	1806	7	0.154	28	3.08	0.31
5133-1@2	156	553	0.16	3.5	43.1	2601	5	0.160	15	2.12	0.14
5133-1@4	66.0	167	0.073	2.5	188	2550	14	0.224	24	1.95	0.32
5133-1@5	87.4	161	0.079	1.9	166	2500	9	0.241	33	1.94	0.33
5133-1@6	402	446	0.23	1.1	26.9	2808	4	0.098	12	2.14	0.10
5133-1@8	67.1	148	0.18	2.2	208	1188	6	0.512	11	2.20	0.57
5133-1@9	56.9	140	0.078	2.5	217	1883	8	0.338	16	2.15	0.34
5133-1@10	91.2	223	0.097	2.4	135	2296	8	0.244	17	2.10	0.24
5133-1@11	55.3	127	0.070	2.3	106	1900	10	0.200	21	2.73	0.34
5133-1@12	109	292	0.16	2.7	721	1606	10	0.329	11	2.56	0.36
5133-1@13	50.1	71.7	0.18	1.4	83.3	967	10	0.672	19	1.34	1.29
5133-1@15	96.4	160	0.080	1.7	21.3	2260	7	0.134	19	2.53	0.20
5133-1@16	116	317	ND[d)]	2.7	495	585	6	0.696	5	1.89	1.28
5133-1@17	66.2	161	0.096	2.4	66.2	1950	8	0.447	14	1.61	0.37
5133-1@18	83.1	241	0.38	2.9	273	723	4	0.643	7	2.13	1.00
5133-1@19	127	376	0.17	3.0	50.7	1935	6	0.173	14	2.79	0.21
5133-1@20	74.1	136	0.077	1.8	36.5	2154	8	0.186	17	2.46	0.24
5133-1@21	85.8	228	0.13	2.7	109	1769	7	0.349	18	2.23	0.37
5133-1@22	147	439	0.38	3.0	743	1041	7	0.401	9	3.38	0.51
5133-1@24	135	166	0.36	1.2	92.7	1139	4	0.536	8	2.12	0.55
5133-1@25	46.0	99.7	0.082	2.2	132	1647	7	0.469	14	1.80	0.45
Sample 11WL59-2											
11WL59-2@01	184	176	0.22	1.0	13.1	1403	4	0.124	13	4.14	0.19
11WL59-2@02	1505	670	70.1	0.45	0.06	25.4	2	0.0511	0.5	249.0	3.7
11WL59-2@03	1621	403	1.03	0.25	2.72	1870	2	0.0608	5	3.38	0.06
11WL59-2@04	4288	1002	199	0.23	0.02	24.1	2	0.0514	0.4	261.7	3.9
11WL59-2@05	2260	259	3.18	0.12	9.55	969	2	0.126	3	5.98	0.12
11WL59-2@06	1878	230	5.58	0.12	0.38	367	2	0.0494	2	17.5	0.3
11WL59-2@07	1713	431	1.18	0.25	3.47	1711	2	0.0570	4	3.71	0.07
11WL59-2@08	532	273	24.7	0.51	0.26	26.1	2	0.0513	0.7	242.0	3.6
11WL59-2@09	2314	262	2.74	0.11	1.45	933	2	0.0519	3	6.85	0.13
11WL59-2@10	1492	444	1.04	0.30	2.83	1721	2	0.0593	4	3.68	0.07
11WL59-2@11	2518	188	2.37	0.08	1.55	1157	2	0.0527	2	5.52	0.10
11WL59-2@12	2016	368	1.37	0.18	2.25	1685	2	0.0574	3	3.77	0.07

a) Sample 5133-1 and samples 11WL59-2 and 11WL60-3 were analysed using the Cameca IMS-1280 SIMS (CASIMS) at the the Institute of Geology and Geophysics, Chinese Academy of Sciences (IGGCAS) and the IMS-1280 SIMS at the State Key Laboratory of Isotope Geochemistry, Guangzhou Institute of Geochemistry, Chinese Academy of Sciences (SKLaBIG GIGCAS), respectively. b) f_{206} is the proportion of common ^{206}Pb in total measured ^{206}Pb. c) $^{206}Pb/^{238}U$ age corrected by ^{207}Pb. D) ND: no data.

Supplementary Table 2 (Continued)

Sample spot	U ppm	Th ppm	Pb ppm	Th/U	f_{206}%[b)]	$^{238}U/^{206}Pb$	±σ(%)	$^{207}Pb/^{206}Pb$	±σ(%)	Age[c)] (Ma)	±σ
Sample 11WL59-2											
11WL59-2@13	1543	155	5.52	0.10	0.55	303	2	0.0486	1	21.2	0.3
11WL59-2@14	3233	853	6.88	0.26	0.62	535	2	0.0479	2	12.0	0.2
11WL59-2@15	1190	135	1.98	0.11	1.11	671	2	0.0543	4	9.50	0.16
11WL59-2@16	5794	933	12.9	0.16	0.24	495	2	0.0476	0.9	13.0	0.2
Sample 11WL60-3											
11WL60-3@1	1290	475	54.3	0.37	0.06	27.7	2	0.0513	0.6	228.3	3.5
11WL60-3@02	1378	518	2.46	0.38	1.84	660	2	0.0534	3	9.67	0.16
11WL60-3@03	1503	235	1.16	0.16	3.49	1482	2	0.0587	5	4.28	0.08
11WL60-3@04	737	281	30.6	0.38	0.08	28.2	2	0.0507	0.8	224.5	3.4
11WL60-3@05	1577	125	10.4	0.08	0.20	168	2	0.0492	1	38.2	0.7
11WL60-3@06	416	306	0.52	0.74	4.65	1120	3	0.0670	10	5.60	0.16
11WL60-3@07	1076	248	0.75	0.23	3.29	1706	2	0.0629	4	3.70	0.08
11WL60-3@08	2075	642	1.51	0.31	2.69	1620	2	0.0550	3	3.93	0.07
11WL60-3@09	2626	437	2.57	0.17	1.94	1155	2	0.0540	3	5.53	0.09
11WL60-3@10	1431	207	3.45	0.15	0.76	461	2	0.0515	2	13.9	0.2
11WL60-3@11	1041	175	1.19	0.17	1.21	1002	2	0.0573	5	6.34	0.12
11WL60-3@12	234	198	0.25	0.85	23.5	1499	4	0.112	12	3.94	0.18
11WL60-3@13	1527	781	67.4	0.51	0.05	27.6	2	0.0519	0.7	229.0	3.4
11WL60-3@14	3293	370	3.11	0.11	1.24	1161	2	0.0500	2	5.52	0.09
11WL60-3@15	679	204	0.53	0.30	5.12	1587	2	0.0685	4	3.95	0.10

Supplementary Table 3 Major element analyses of titaniferous magnetite (TM), ilmenite (Ilm) and F-rich mica (FM) from the Dongyue Lake lavas (in wt.%)

sample spot	5123_18	5127_3	5133_3	5124_1	5123_8	5123_15
mineral	TM	TM	TM	TM	Ilm	Ilm
SiO_2	0.31	0.44	0.20	0.17	0.25	0.12
TiO_2	9.16	9.34	9.21	7.83	48.80	50.35
Al_2O_3	2.61	1.30	2.39	1.63	0.47	0.45
CaO	0.00	0.00	0.00	-	-	-
MnO	0.36	0.58	0.50	0.47	0.57	0.46
MgO	1.08	0.27	1.81	1.23	3.94	3.51
FeO	83.35	84.58	82.54	82.33	46.04	46.79
Cr_2O_3	0.08	0.12	0.27	0.16	0.05	0.07
NiO	0.00	0.00	0.01	-	0.01	
Na_2O	0.06	0.06	0.15	0.14	-	-
K_2O	0.00	0.00	0.00	-	-	-
Cl	0.00	0.01	0.00	0.00	-	0.00
F	0.00	0.00		-	-	-
Total	97.01	96.68	97.07	93.96	100.12	101.74

Supplementary Table 3 (continued)

sample spot	5124-1_6	5124-1_8	5124-1_11	5124-2_4	5124-2_5	5133-1_1
mineral	FM	FM	FM	FM	FM	FM
SiO_2	39.76	39.43	39.38	39.44	38.14	38.49
TiO_2	2.99	2.93	3.07	3.05	3.18	3.89
Al_2O_3	11.22	11.82	11.90	12.00	12.24	12.36
FeO	10.27	9.42	9.27	10.55	9.12	10.51
MnO	0.08	0.09	0.07	0.10	0.08	0.09
MgO	20.00	21.28	20.98	20.32	20.58	19.28
CaO	0.01	0.04	0.01	0.02	0.03	0.02
Na_2O	0.51	0.58	0.53	0.60	0.59	0.61
K_2O	9.60	9.77	9.69	9.73	9.17	8.88
F	4.97	4.78	4.77	4.64	4.64	4.61
Cl	0.33	0.32	0.32	0.27	0.26	0.16
Total	99.72	100.46	99.98	100.71	98.03	98.91

Supplementary Table 4 Calculated degrees of melting ($F^{a)}$)

Methods	Qiangtang		Songpang-Ganzi	Central Kunlun	
	non-adakitic trachyandesites and dacites	Adakitic rhyolites	strongly peraluminous rhyolites	non-adakitic trachyandesites	Adakitic trachyandesites
F (vol.%) by Method 1[b)]	22 %[c)]	10 %[c)]	9 %[c)]	—	—
F (vol.%) by Method 2[b)]	19 %[c)]	8 %[c)]	10 %[c)]	16 %[c)]	21 %[c)]

a) *F* is the melt fraction or proportion. **b**) Methods 1 and 2 are based on simple batch melting models. Method 1: calculated from estimates of Rb/Sr in the source from combined Nd and Sr isotopes and the measured Rb/Sr of the samples, and appropriate distribution coefficients for the different minerals. Method 2: calculated from the rare earth element (REE) contents of the samples and appropriate distribution coefficients of different minerals. The detailed models and equations are presented in Supplementary Method 1. **c**) Overall the results from the two methods (Supplementary Table 4) show that the crustal melts from central and northern Tibet reflect ~8−22% partial melts.

Supplementary Table 5 The calculated degrees of partial melting for Pliocene-Quaternary felsic volcanic lavas from central and northern Tibet in terms of Rb/Sr

Sample	Sm	Nd	$^{147}Sm/^{144}Nd$	$^{143}Nd/^{144}Nd$	Age (Ma)	T_{DM} Nd (Ma)[d)]	Rb	Sr	$^{87}Rb/^{86}Sr$	$^{87}Sr/^{86}Sr$	model (Stage 1) $^{87}Rb/^{86}Sr$[d)]	model $(Rb/Sr)_{source\ rocks}$[d)]	$F_{melting\ degree}$ (vol. %)[f)]	$(Rb/Sr)_{calculated}$	$(Rb/Sr)_{sample}$
5123-2	13.3	98.4	0.0824	0.51227	2.3	1023	226	498	1.31	0.70965	0.53	0.18	18	0.46	0.45
5124-2	14.4	110	0.0799	0.51230	2.3	973	242	528	1.33	0.70955	0.55	0.19	19	0.46	0.46
5126-1	15.5	117	0.0804	0.51225	2.3	1035	275	506	1.57	0.70969	0.53	0.18	12	0.54	0.54
5127-3	14.8	111	0.0806	0.51227	2.3	1007	240	445	1.56	0.70971	0.55	0.19	13	0.54	0.54
5133-1	15.2	117	0.0790	0.51228	2.3	989	252	495	1.47	0.70971	0.56	0.19	16	0.50	0.51
C1601-1	14.2	88.5	0.0971	0.51234	2.5	1065	258	486	1.53	0.70976	0.52	0.18	12	0.53	0.53
C1603-1h	13.7	87.0	0.0955	0.51237	2.5	1010	250	488	1.47	0.70943	0.52	0.18	13	0.52	0.51
11WL59-2	14.7	102	0.0872	0.51226	3.8	1074	172	639	0.778	0.70953	0.50	0.17	32	0.27	0.27
11WL59-3	13.8	94.1	0.0892	0.51228	3.8	1070	170	633	0.777	0.70956	0.51	0.18	33	0.27	0.27
11WL61-3	13.2	89.1	0.0899	0.51228	3.8	1077	173	648	0.773	0.70953	0.50	0.17	32	0.27	0.27
11WL60-3	14.4	99.0	0.0886	0.51226	3.8	1085	174	658	0.765	0.70954	0.50	0.17	33	0.26	0.26
MB1-1	14.2	95.1	0.0901	0.51229	2.5	1066	219	676	0.936	0.70945	0.50	0.17	20	0.33	0.32
MB4	14.5	94.6	0.0928	0.51231	2.5	1056	208	582	1.03	0.70937	0.50	0.17	16	0.35	0.36
Average[a)]												**0.18**	**22**	**0.40**	**0.41**
D7535C1[b)]	5.31	38.0	0.0848	0.51228	3.2	1025	179	586	0.885	0.70972	0.54	**0.19**	**10**	**0.30**	**0.31**
2303	6.32	38.3	0.1003	0.51228	3.0	1171	425	90.9	13.5	0.71497	0.76	0.26	13	4.58	4.67
2511-1	11.6	71.2	0.0991	0.51234	1.5	1081	476	71.5	19.3	0.71301	0.70	0.24	6	6.58	6.65
Average[c)]												**0.25**	**9**	**5.57**	**5.66**

a) Average compositions for non-adakitic trachyandesites and dacites in the Qiangtang block, central Tibet; b) Adakitic rhyolites in the Hengliangh area, Qiangtang block of central Tibet; c) Average compositions for strongly peraluminous rhyolites in the Songpan-Ganzi block; d) The calculations are after from Dhuime et al.[18]; f) The degree of partial melting is calculated by Equation 2, which is from Supplementary Method 1.

Supplementary Table 6 Other data for calculating the degrees of melting for the Qiangtang non-adakitic rocks

	Mineral[a)]	Opx	Cpx	Grt	Amp	Bt	Pl	sum
	Mineral proportion	0.20	0.35	0.10	0.03	0.02	0.30	1.00
Rb	distribution coefficient [b)]	0.022	0.031	0.042	0.176	3.53	0.071	
Sr	distribution coefficient [b)]	0.017	0.120	0.012	0.460	0.120	1.83	

a) Opx: orthopyroxene; Cpx: clinopyroxene; Grt: garnet; Amp: amphibole; Bt:biotite; Pl: plagioclase. b) Distribution coefficients for basalts and andesites are after Hanson[19] and Klein et al.[20].

Supplementary Table 7 Other data for calculating the degrees of melting for the Qiangtang adakitic rocks

	Mineral[a)]	Opx	Cpx	Grt	Amp	Bt	Pl	sum
	Mineral proportion	0.04	0.50	0.40	0.01	0.01	0.05	1.00
Rb	distribution coefficient[b)]	0.022	0.031	0.042	0.176	3.53	0.071	
Sr	distribution coefficient[b)]	0.017	0.120	0.012	0.460	0.120	1.83	

a) Opx: orthopyroxene; Cpx: clinopyroxene; Grt: garnet; Amp: amphibole; Bt:biotite; Pl: plagioclase. b) Distribution coefficients for basalts and andesites are after Hanson[19] and Klein et al.[20].

Supplementary Table 8 Other data for calculating the degrees of melting for the Songpan-Ganzi rhyolites

	Mineral [a)]	Opx	Cpx	Grt	Amp	Bt	Pl	Kf	Sum
	Mineral proportion	0.01	0.02	0.04	0.04	0.01	0.85	0.03	1.00
Rb	distribution coefficient[b)]	0.003	0.032	0.009	0.045	3.26	0.041	0.366	
Sr	distribution coefficient[b)]	0.009	0.516	0.015	0.280	0.120	4.40	3.87	

a) Opx: orthopyroxene; Cpx: clinopyroxene; Grt: garnet; Amp: amphibole; Bt:biotite; Pl: plagioclase; Kf: K-feldspar. b) Distribution coefficients for dacites and rhyolites are after Hanson[19] and Klein et al.[20].

Supplementary Table 9 Selected partition coefficients for trace elements between melts and minerals used in Equation 1 [a)]

Minerals	La	Sm	Yb	Lu	References
amphibole	0.37	2.01	2.1	1.7	Klein et al.[20]
garnet	0.39	2.04	43.8	39.8	Irving & Frey[21]
plagioclase	0.38	0.165	0.09	0.092	Nash & Crecraft[22]
biotite	5.71	2.12	1.47	1.62	Nash & Crecraft[22]
clinopyroxene	1.11	5.23	6.37	5.93	Nash & Crecraft[22]
orthopyroxene	0.78	1.6	2.2	2.25	Nash and Crecraft[22]
K-feldspar	0.08	0.025	0.03	0.033	Nash & Crecraft[22]

a) Equation 1 is from Supplementary Method 1.

Supplementary Table 10 Major (wt.%) and trace (ppm) data of starting materials (source rocks) for the partial melting calculations

sample	LBT20[a)]	GZ-47[b)]	LH01-8[c)]
rock	Granulite xenolith	sedimentary rocks	Garnet-bearing amphibolite xenolith
SiO_2	52.67	48.64	47.40
TiO_2	1.03	0.69	1.62
Al_2O_3	21.74	15.29	10.93
Fe_2O_3	5.08	0.45	3.67
FeO	2.58	6.04	3.67
MnO	0.08	0.09	0.21
MgO	3.06	3.65	12.3
CaO	5.62	10.18	8.80
Na_2O	4.2	1.09	1.25
K2O	2.51	3.04	2.07
P_2O_5	0.21	0.15	0.200
La	70.8	40.0	22.9
Ce	144	76.8	19.2
Pr	14.3	8.82	6.01
Nd	50.0	30.7	32.0
Sm	8.00	6.02	8.68
Eu	1.36	1.20	2.20
Gd	7.27	5.21	8.72
Tb	0.87	0.77	1.62
Dy	4.54	4.48	10.5
Ho	0.87	0.90	2.15
Er	2.19	2.72	6.47
Tm	0.31	0.40	1.03
Yb	2.02	2.56	6.69
Lu	0.32	0.40	0.93

a) Granulite xenoliths from Cenozoic volcanic lavas in the Qiangtang Block are from Lai et al. [17], they have Nd-Sr compositions similar to the Qiangtang non-adakitic trachyandesites and dacites, and they are assumed to represent the source rocks of the Qiangtang and Central Kunlun non-adakitic trachyandesites and dacites; **b**) Pre-Jurassic clastic sedimentary rocks in the Songpan-Ganzi Block are from Chen et al.[23], they have Nd compositions similar to northern Tibet strongly peraluminous rhyolites, and are assumed to represent the source rocks of the Songpan-Ganzi strongly peraluminous rhyolites; **c**) Garnet-bearing amphibolite xenoliths from Cenozoic volcanic lavas in the eastern Qiangtang Block are from Wang et al.[24], and are assumed to represent the sources of the Qiangtang and Central Kunlun adakitic rhyolites and trachyandesites.

Supplementary Table 11 The calculated degrees of partial melting for selected Pliocene-Quaternary felsic volcanic lavas from central and northern Tibet in terms of average REE contents

Block	Rocks or Model calculations	Average values or melting degree (F, vol%) [a, b]	La (ppm)	Sm (ppm)	Yb (ppm)	Lu (ppm)	Selected source samples
Qiangtang	non-adakitic trachyandesites and dacites	19 samples [a]	154	13.5	1.69	0.24	LBT20 [c]
	Model calculation	F = 19	155	12.5	1.66	0.28	
	Adakitic rhyolites	2 samples [a]	78.3	4.91	0.88	0.13	LH01-8 [c]
	Model calculation	F = 8	77.9	9.44	0.88	0.13	
Songpan-Ganzi	Strongly peraluminous rhyolite	8 samples [a]	32.9	5.06	0.48	0.069	GZ-47 [c]
	Model calculation	F = 10	32.8	5.27	0.47	0.080	
Central Kunlun	non-adakitic trachyandesites	8 samples [a]	169	16.7	1.39	0.20	LBT20 [c]
	Model calculation	F = 16	169	13.2	1.37	0.24	
	Adakitic trachyandesites	2 samples [a]	58.3	7.71	1.06	0.15	LH01-8 [c]
	Model calculation	F = 21	58.2	9.33	1.01	0.15	

a) The data are from Supplementary Dataset 1; b) The data and results of model calculations on partial melting using the REE contents of rocks based on Equation 1, which is from Supplementary Method 1; c) The samples are from Supplementary Table 10.

Supplementary Methods
Batch melting models

The simple batch melting model[25] was used as follows:

$$C_i^l / C_i^o = 1/(D^{s/l} \times (1-F) + F) \ldots\ldots (Equation\ 1)$$

F is the melt proportion or fraction; C_i^o is the content of element i in original solid parent material; C_i^l is the content of element i in liquid material; D is the bulk distribution coefficients for element i.

To evaluate the degree of partial melting, the Rb/Sr ratio in the source rocks (*model* $(Rb/Sr)_{source\ rocks}$) can be estimated from Nd and Sr isotopes[18], and the relationship between the $(Rb/Sr)_{calculated}$ of the melts and F with the following equation:

$$(Rb/Sr)_{calculated} = model\ (Rb/Sr)_{source\ rocks} \times (D^{Sr} \times (1-F) + F)/(D^{Rb} \times (1-F) + F) \ldots\ldots (Equation\ 2)$$

D^{Rb} and D^{Sr} are the bulk distribution coefficients for Sr and Rb, and the Rb/Sr ratios of the magmas ($(Rb/Sr)_{sample}$) are those measured on the whole rock samples. The preferred melt fraction is that required for $(Rb/Sr)_{calculated}$ to equal $(Rb/Sr)_{sample}$. The calculated degrees of partial melting for selected Pliocene−Quaternary felsic volcanic lavas in central and northern Tibet based on Equation 2 are summarized in Supplementary Tables 5–8 (Method 1).

The degrees of partial melting were also calculated using rare earth element (REE) contents of rock samples based on Equation 1. The calculated degrees of partial melting for the Pliocene−Quaternary volcanic lavas in central and northern Tibet are shown in Supplementary Tables 9–11 (Method 2).

Supplementary References

1. Jin, C. H. *et al.* Geochemical features of Neogene volcanic rocks in the Shawotan-Hongyuanquan area of northern Qiangtang in Tibet and their tectonic implications. *Geol. Explor.* 46, 1061–1070 (2006).

2. Wang, Q. *et al.* Eocene melting of subducting continental crust and early uplifting of central Tibet: Evidence from central–western Qiangtang high-K calc-alkaline andesites, dacites and rhyolites. *Earth Planet. Sci. Lett.* 272, 158–171 (2008).

3. Wei, J.Q., Wang, J.X., Niu, Z.J. The Cenozoic volcanic rocks from the Chibuzhang Lake area in the Qiangtang area. *Sediment. Geol. Tethy. Geol.* 24, 16–21 (2004).

4. Wang Q. *et al.* Crustal melting and flow beneath northern Tibet: Evidence from Mid-Miocene to Quaternary strongly peraluminous rhyolites in southern Kunlun Range. *J. Petrol.* 53, 2523–2566 (2012).

5. Jolivet, M. *et al.* Neogene extension and volcanism in the Kunlun Fault Zone, northern Tibet: New constraints on the age of the Kunlun Fault. *Tectonics* 22, 1052 (2003).

6. Burchfiel, B. C. *et al.* Geology of the Ulugh Muztagh area, northernTibet. *Earth Planet. Sci. Lett.* 94, 57–70 (1989).

7. McKenna, L.W & Walker, J.D. Geochemistry of crustally derived leucocratic igneous rocks from the Ulugh Muztagh area, northern Tibet and their implications for the formation of the Tibetan Plateau. *J. Geophys. Res.* 95, 21483–21502 (1990).

8. Li, J. D., Bai, D. Y., Wang, X. H. Ages of volcanic rocks and planation surface in the Canmei Mountain Area, northern Tibet. *Geol. Bull. China* 23, 670–675 (2004).

9. Zhao, Z. M. *et al*. Geochemical characteristics and petrogenesis of volcanic rocks since the

Neogene in the Bayankala and east Kunlun region, northern Tibetan Plateau. *Geochim.* 38, 205–230 (2009).

10. Chen, B. H. *et al*. Characteristics and tectonic environments of quaternary volcanic rocks in Jindingshan, northern Tibetan Plateau. *Acta Petrol. Mineral.* 22, 125–130 (2003).
11. Zhang, X. X. & Chen, B. H. Geology-geochemistry and Genesis of Quaternary Volcanic Rocks in the Feiyunshan Area, West Kunlun. *J. East China Inst. Technol.* 28, 216–222 (2005).
12. Watson, E. B. & Harrison, T. M. Zircon saturation revisited: temperature and composition effects in a variety of crustal magma types. *Earth Planet. Sci. Lett.* 64, 295–304 (1983).
13. Sun, S. S. & McDonough,W. F. Chemical and isotopic systematics of oceanic basalts: Implications for mantle composition and processes. In: Saunders, A. D. & Norry, M. J. (eds) *Magmatism in the Ocean Basins*. *Geological Society, London, Special Publications* 42, 313–345 (1989).
14. Defant, M. J. & Drummond, M. S. Derivation of some modern arc magmas by melting of young subducted lithosphere. *Nature* 347, 662–665 (1990).
15. Rapp, R. P., Shimizu, N. & Norman, M. D. Growth of early continental crust by partial melting of eclogite. *Nature* 425, 605–609 (2003).
16. Xiong, X.L., Adam, J. & Green, T.H. Rutile stability and rutile/melt HFSE partitioning during partial melting of hydrous basalt: Implications for TTG genesis. *Chem. Geol.* 218, 339–359 (2005).
17. Lai, S.C. & Qin, J.F. Petrology and geochemistry of the granulite xenofiths from Cenozoic Qiangtang volcanic field：Implication for the nature of the lower crust in the northern Tibetan plateau an d the genesis of Cenozoic volcanic rocks． *Acta Petrol. Sin.* 24, 325–336 (2008).

18. Dhuime, B. *et al*. Emergence of modern continental crust about 3 billion years ago. *Nat. Geosci.* 8, 552–555 (2015).

19. Hanson, G. N. Rare earth elements in petrogenetic studies of igneous system. *Am. Rew. Earth Plan. Sci.* 8, 371–406 (1980).

20. Klein, M. et al. Partitioning of high field strength and rare earth elements between amphibole and quartz-dioritic to tonalitic melts: an experimental system. *Chem. Geol.* 138, 257–271 (1997).

21. Irving, A. J. & Frey, F. A. Distribution of trace-elements between garnet megacrysts and host volcanic liquids of kimberlitic to rhyolitic composition. *Geochim. Cosmochim. Acta* 42, 771–787 (1978).

22. Nash, W. P. & Crecraft, H. R. Partition coefficients for trace elements in silicic magmas. *Geochim. Cosmochim. Acta* 49, 2,309–2,322 (1985).

23. Chen, Y. L. *et al*. Elemental and Sm-Nd isotopic geochemistry of clastic sedimentary rocks in the Garzê-Songpan and Longmen Mountains. *Geol. China* 33, 109–118 (2006).

24. Wang, Q. *et al*. Cenozoic K-rich adakitic volcanic rocks in the Hohxil area, northern Tibet: Lower crustal melting in an intracontinental setting. *Geology* 33, 465−468 (2005).

25. Shaw, D. M. Trace element fractionation during anatexis. *Geochim. Cosmochim. Acta* 34, 237−243 (1970).
